# Deciphering transcriptional plasticity in pancreatic ductal adenocarcinoma reveals alterations in sensory neuron innervation

**DOI:** 10.1002/1878-0261.70233

**Published:** 2026-03-18

**Authors:** Elena Genova, Michele Montrone, Uday Rangaswamy, Francesco Diversi, Irene Schiavo, Denise Ferrarini, Roberta Di Florio, Irene Longo, Michele Coscia, Nicola Zamboni, Giorgia Demontis, Lisa Veghini, Vincenzo Corbo, Remo Sanges, Paul Heppenstall

**Affiliations:** ^1^ Institute for Maternal and Child Health‐ IRCCS “Burlo Garofolo” Trieste Italy; ^2^ Scuola Internazionale Superiore di Studi Avanzati Trieste Italy; ^3^ Department of Engineering for Innovation Medicine University of Verona Italy

**Keywords:** pancreas, pancreatic ductal adenocarcinoma, sensory neurons, single‐cell RNA sequencing, tumor microenvironment

## Abstract

Neuronal innervation of the pancreas has historically been characterized using marker‐based classification and physiological studies, but its transcriptomic landscape remains only partially explored. A detailed molecular profile of pancreatic sensory neurons could provide insights into their role in health and disease, particularly in pancreatic ductal adenocarcinoma (PDAC), where neural remodeling influences tumor progression and pain signaling. Wild‐type and PDAC mice were injected with the retrotracer Fast Blue into pancreatic or cancerous tissue. Dorsal root ganglia were dissociated, and Fast Blue‐positive sensory neurons were isolated, lysed, and analyzed using single‐cell RNA sequencing. Data were validated using immunofluorescence, organoid cultures and qPCR. We performed transcriptomic profiling of sensory neurons innervating the pancreatic head and tail under normal and cancer conditions. Our analysis identified neurofilament‐containing neurons as the predominant sensory subtype in both contexts, while non‐peptidergic neurons were underrepresented in tumor‐associated innervation. Differential gene expression analysis revealed a unique subset of genes upregulated in sensory neurons innervating pancreatic tumors, many linked to mitochondrial activity. Further validation also revealed the presence of transcripts transferred via extracellular vesicles (including the Pdx1‐CreERT2 transgene from the KPC mouse model), suggesting a novel mechanism of tumor–neuron interaction. Our findings provide a detailed characterization of pancreatic and pancreatic ductal adenocarcinoma sensory innervation. We identified tumor‐derived RNA within sensory neurons in the PDAC mouse model, suggesting an extracellular vesicle–mediated RNA transfer mechanism that may remodel sensory signaling and open new prospects for diagnostic and therapeutic innovation in PDAC.

**Impact statement**

Transcriptomic profiling of pancreatic sensory neurons reveals shifts in neuronal populations, tumor‐specific mitochondrial gene upregulation, and potential extracellular vesicle–mediated transcript transfer. Circulating tumor transcripts in KPC mice provide a reference for pancreatic innervation, tumor–nerve interactions, and therapeutic targets.

## Graphical abstract

Pancreatic sensory neurons innervating healthy and PDAC tissue were retrogradely labeled and profiled by single‐cell RNA sequencing. Tumor‐associated innervation showed a dominant neurofilament‐positive subtype, altered mitochondrial gene signatures, and reduced non‐peptidergic neurons. Transcriptomic and imaging analyses revealed tumor‐derived RNA within sensory neurons, supporting extracellular vesicle–mediated tumor–neuron communication in pancreatic cancer.

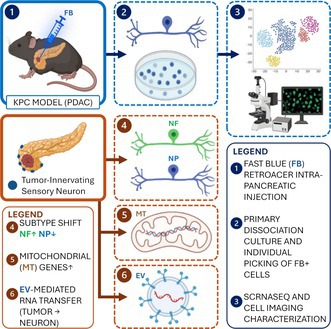

**Abbreviations**
5′UTR: untranslated region
DRG: dorsal root ganglia
EVs: extracellular vesicles
FB: Fast Blue
IF: immunofluorescence
KPC: Kras TrP53 Cre
LncRNA: long non‐coding RNA
NF: neurofilament containing
NP: non‐peptidergic
PDAC: pancreatic ductal adenocarcinoma
PEP: peptidergic
ScRNA‐seq: single‐cell RNA sequencing
TH: tyrosine hydroxylase
WT: wild type

## 1 Introduction

The pancreas interacts with the nervous system through an intricate network of nerves that derive from the sympathetic, parasympathetic, and sensory nervous systems [1]. Studies show that these systems preferentially innervate different regions of the pancreas, contributing to their distinct yet interdependent roles in pancreatic function which are not yet fully understood.

In the healthy pancreas, this innervation helps regulate digestive enzyme secretion, blood flow, and inflammatory responses, maintaining homeostasis by ensuring appropriate responses to physiological needs, such as digestion or blood sugar regulation [2]. The contribution of nerves in the context of cancer pathophysiology is also significant, and cancer severity correlates with innervation density [3, 4]. As the disease advances, increased nerve density and neural remodeling become hallmark features of the tumor microenvironment.

Previous research has shown that denervation of sympathetic neurons inhibits tumor progression [5], while transection of the parasympathetic vagal input to the pancreas accelerates progression of pancreatic ductal adenocarcinoma (PDAC), indicating that sympathetic and parasympathetic inputs play opposing roles [6, 7]. There is also a prominent cross‐talk between innervating sensory neurons and cancer cells that drives inflammation, promotes tumor progression, and underlies the debilitating pain reported by PDAC patients [8]. Ablation of nociceptors in experimental models of PDAC and PanIN not only reduces nociceptive behavior but also significantly delays tumorigenesis and prolongs survival, highlighting the importance of sensory neurons in sustaining cancer progression [9, 10]. While providing the first evidence for an interaction between sensory neurons and cancer cells, these studies raise questions as to the molecular mechanisms of the interaction and the contribution of other types of sensory neurons, beyond nociceptors, to the circuit.

Sensory neurons can be classified based on their anatomical, functional, and neurochemical properties [11, 12, 13]. Early classifications focused on broad distinctions between nociceptive and non‐nociceptive neurons, such as differences in cell soma size, degree of myelination of their axons, their termination patterns, and thresholds of responses to defined stimuli. With advancements in transcriptomic profiling, more refined classifications emerged. One of the early landmark studies in this area was conducted by Usoskin *et al*. [14], who used single‐cell RNA sequencing of mouse Dorsal Root Ganglion (DRG) neurons to categorize sensory neurons into several distinct subtypes. This molecularly driven nomenclature categorizes sensory neurons into four main types distinguished by gene expression and putative functional roles: NF neurons express neurofilament heavy chain and are associated with myelinated mechanoreceptors and proprioceptors, PEP neurons express neuropeptides like substance P and calcitonin gene‐related peptide and were previously classified as peptidergic nociceptors, NP neurons correspond to non‐peptidergic nociceptors, and TH neurons show expression of tyrosine hydroxylase and likely function as unmyelinated low threshold mechanoreceptors. More recently, similar studies with increased resolution have been conducted across various species to refine the classification further [15, 16, 17, 18]. This framework has facilitated the exploration of sensory neuron function in chronic pain, inflammation, and disease progression, including cancer.

Here, we provide a molecular classification of sensory neuron populations innervating healthy and PDAC pancreas, at a single neuron level. We delineate which sensory neuron subtypes innervate the pancreas and show how this is altered during PDAC. Our analysis also provides a gene expression profile of these neurons, revealing transcriptional plasticity within neurons as PDAC progresses. Changes in the molecular profile of pancreas‐innervating sensory neurons likely contribute to the altered crosstalk between neurons and pancreatic cells during tumorigenesis. Thus, such a reference dataset may reveal novel molecular strategies to interfere with this crosstalk and delay cancer progression.

## 2 Materials and methods

### 2.1 Animals used for scRNA‐seq and EVs isolation

All animal procedures complied with EU Directive 2010/63/EU, Italian law (Decree 26/14), and were approved by SISSA's Ethical Committee under project 22DAB16 (authorization n. 92/2021‐PR), authorized by the Italian Ministry of Health per EU Recommendation 2007/526/CE.

The tamoxifen‐inducible Kras^LSL‐G12D^; p53^LoxP^; Pdx1‐CreER (KPC) PDAC mouse model (Charles River, #032429) was used. Tumor induction was achieved by orally administering 6 mg tamoxifen in corn oil to lactating mothers on postnatal days 0, 1, 2, and 4. Mice were sacrificed at 8–9 weeks to assess pancreatic intraepithelial neoplasia (PanIN), and at 11–13 weeks for invasive PDAC analysis.

For scRNA‐seq, adult C57Bl/6J mice (≥ 7 weeks old) served as controls. For EV isolation, WT siblings lacking the Kras^LSL‐G12D^ mutation were used. Mice were housed under a 12/12 h light–dark cycle with enriched environments and *ad libitum* access to food and water. Newborns received a high‐energy diet (5LJ5, PicoLab^®^) to support normal growth. Only adult mice (8–15 weeks old, both sexes) were included in experiments.

### 2.2 Retrograde labeling

To identify sensory neurons innervating healthy versus tumoral pancreatic tissue, retrograde labeling with 2% Fast Blue (FB, Polysciences #17740‐1, Warrington, PA, USA) was performed in C57Bl/6J and KPC mice aged 8–13 weeks. Mice were anesthetized with 4% isoflurane in 100% O_2_ (1.0 L·min^−1^) for induction and maintained under 2.0% isoflurane during surgery. Adequate anesthesia was confirmed via the absence of the pedal reflex. A total of 4 μL of FB solution was injected into either healthy pancreatic tissue or tumor (4–5 injections of 1 μL each) using a Hamilton syringe. The abdominal cavity was closed with sterile, absorbable surgical sutures (FPGLA1CN, Vetsuture). DRG from adult KPC and C57Bl/6J mice (8–13 weeks old) were collected 1 week post injection and were prepared as previously described [19].

### 2.3 Primary DRG cell culture

DRG neurons from adult KPC and C57Bl/6J mice (8–12 weeks old) were prepared as previously described. Briefly, adult mice (≥ 7 weeks old) were euthanized in a CO_2_ chamber and the spinal column rapidly removed. All DRG neurons were removed and placed in PBS. Sensory ganglia were enzymatically digested in Collagenase type IV (10 ng·μL^−1^, Sigma‐Aldrich C5138, St. Louis, MO, USA) resuspended in 1 mL of DMEM‐GlutaMAXTM (31966047, Life Technologies‐Gibco, Waltham, MA, USA) for 26 min at 37 °C. Then, cells were centrifuged and incubated with 1 mL of 0.05% Trypsin EDTA (Gibco 25300‐054, Grand Island, NY, USA) for 22 min at 37 °C. Trypsin was inactivated with 500 μL of DMEM‐GlutaMAXTM supplemented with 10% FBS (ECS0180L, Euroclone) and 1% pen/strep. Cell debris was removed by passing cell suspension through a 100 μm filter (BD™, 340632). The cell suspension was pelleted and resuspended in DMEM‐GlutaMAX™ (Gibco, 31966‐047) supplemented with 10% FBS (Euroclone, ECS0180L) and 1% pen/strep (Euroclone, ECB3001D, Pero, MI, Italy) and plated in a droplet of growth medium on a 35 mm glass coverslip (MatTek, Milan, MI, Italy) precoated with poly‐L‐lysine (100 μg·mL^−1^) and laminin (20 μg·mL^−1^). Cells were maintained at 37 °C and 5% CO_2_ in a humidified incubator. For the scRNA sequencing experiment 6 and 12 independent biological replicates were taken for tumor and healthy group, respectively.

### 2.4 Immunofluorescence

Thoracic and lumbar DRG were collected from C57Bl/6J and KPC mice (female and/or male) 1 week after retrotracer injection into the pancreas. Tumor innervation was not analyzed after week 14 due to technical limitations related to tumor size (> 1.5 × 1.5 cm), which prevented accurate retrotracer injection. Single‐cell suspensions were prepared as described above and plated onto glass coverslips overnight. Cells were washed with PBS, fixed with 4% PFA for 10 min, and permeabilized using 0.05% Triton X‐100. After additional washes, samples were blocked with 3% goat serum in 0.01% Tween‐20 for 30 min. Cells were then incubated overnight at 4 °C with primary antibodies diluted in blocking solution. The primary antibodies used included anti‐NF200, anti‐TH, and anti‐SubP. Samples were incubated with secondary antibodies and Isolectin GS‐IB4 Alexa Fluor™ 647 for 2 h at 4 °C after two ice‐cold PBS washes. *Z*‐stack images were collapsed by maximal intensity projection and analyzed using Fiji (ImageJ). A fixed threshold for signal detection was empirically defined to optimize signal‐to‐noise ratio and applied uniformly across all images. For co‐localization analysis of Fast Blue–positive neurons with specific neuronal markers, images were thresholded to generate binary masks, and true overlap was identified using Boolean AND operations. Binary outputs were refined by removal of small particles to ensure specificity of signal overlap. Cells positive for more than one neuronal marker were counted separately and excluded from subtype quantification. Four fields per animal were analyzed in DRG cultures from 3 WT and 2 KPC mice, with an average of 37–38 Fast Blue–positive cells per field in both groups. Z‐stack images were captured using a Nikon C2+ confocal microscope with a 10× objective, comprising 5 optical slices (100 μm total thickness, 20 μm intervals). Two fields per animal were analyzed in ImageJ, followed by manual counting of overlapping signals with the “Cell Counter” plugin [20]. Immunostaining employing the same antibodies was performed on pancreas sections from both WT and KPC mice.

### 2.5 Sensory neuron subpopulation marker immunostaining on pancreatic slices

Pancreata dissected from both tamoxifen‐inducible Kras^LSL‐G12D^; p53^LoxP^; Pdx1‐CreER (KPC) mice PDAC mouse model (Charles River, #032429, Wilmington, MA, USA) and C57BL/6J were fixed with 4% PFA at RT for 4 h and let sinking overnight in a 30% Sucrose solution at the bottom of a 15 mL Falcon tube. Prior to cryostat cutting (Cryostat OTF5000, Bio Optica, Milano, MI, Italy), samples were embedded in O.C.T mounting media for cryotomy (05‐980, Bio Optica) and left to harden at −80 °C for 15 min. Cryostat settings were as follows: −12 °C for sample, −25 °C for chamber, fine 30 micrometers, trimming 40. Each slice was placed in a glass slide (76 × 26mm, SuperFrost Plus, J1800AMNZ, MENZEL‐GLÄSER). The slices were kept on a wet support for the entire duration of the staining. Samples were incubated with blocking solution BSA 1% + Triton™ X‐100 0.3% (T8787, Sigma‐Aldrich, St. Louis, MI, USA) for 2 h at room temperature. Primary antibody staining occurred overnight at 4 °C, 1 : 200 in blocking solution. The next day, 3 washes in PBS 1x were performed and were followed by incubation with secondary antibody 1 : 500 for 2 h at room temperature. After two washes in PBS 1×, coverslips (BB024060A1, MENZEL‐GLÄSER) were mounted over the slides with ProLong™ Diamond Antifade Mountant (P36970, ThermoFisher, Waltham, MA, USA). The primary antibodies used were mouse anti‐NF200 (Merck, MAB5266, Kenilworth, NJ, USA), rabbit anti‐TH (Thermofisher, PA5‐18372), rabbit anti‐βTubulin III (Sigma, T2200, St. Louis, MI, USA). For detection, goat 488‐conjugated Alexa secondary antibody against mouse (Invitrogen, A11029, Waltham, MA, USA) and goat 594‐conjugated Alexa secondary antibody against rabbit (Invitrogen, A11032) were used. For isolectin B4 staining, Isolectin GS‐IB_4_ From *Griffonia simplicifolia*, Alexa Fluor™ 647 Conjugate (Thermofisher, 1 : 500, I32450) was added to the secondary antibodies mix.

### 2.6 Organoid culture

The murine cell lines mNP (WT) and KPC were established and provided by the Vincenzo Corbo's laboratory. Procedures used to isolate normal and neoplastic pancreatic cells were adapted from Boj et al. [21]. Briefly, pancreatic tissues from wild‐type and KPC mice were enzymatically digested with 0.012% (w/v) collagenase XI (Sigma) and 0.012% (w/v) dispase (GIBCO) in DMEM media containing 1% FBS (GIBCO) for up to 2 h and resulting cell clusters seeded in growth factor reduced matrigel (corning) overlaid with organoid culture medium. Cell line identity was confirmed within the past 3 years by PCR genotyping for floxed alleles. All experiments were performed using mycoplasma‐free cells. Normal pancreas (mNP ‐ WT) and cancer organoids (KPC) were cultured following the Tuveson Laboratories [21] guidelines for murine organoids. Briefly, organoids (four standard domes/well) were cultured with 2 mL of feeding medium for 3 days in a 6‐well plate at 37 °C with 5% CO_2_.

### 2.7 Mitochondria staining in DRG neurons

To validate gene ontology findings on differentially regulated genes, mitochondrial abundance and distribution were assessed in DRG innervating either normal or tumor‐bearing pancreas. Fast Blue retrograde tracing was used to label DRG in one KPC mouse and a sibling WT control. Single‐cell suspensions were prepared from thoracic and lumbar DRG. After 24 h of incubation, cells were stained with 200 nm MitoRed dye (53271, Sigma Aldrich) for 30 min at 37 °C. Following washes, cells were processed for IF (NF, NP, PEP, TH). Imaging was performed using a Nikon C2+ confocal microscope with 20–24 optical sections per sample at 0.2 μm resolution. In total, 80 neurons were analyzed—40 from each condition—ensuring representation of each neuronal cluster type.

### 2.8 Histology

The tissue was fixed in 10% formalin for 24–48 h based on tissue dimension at room temperature. After washing with PBS, the fixed tissue was dehydrated with ethanol and xylene. The tissue was incubated in liquid paraffin (65 °C) two times for 30 min each and then embedded in paraffin and let it harden at 4 °C. 4 μm sections of mouse tissues were stained with Hematoxylin and Eosin and scanned at 40X magnification using Aperio GT450 DX (Leica). The description of the exocrine pancreatic lesions followed the criteria defined in Hruban *et al*. [22]. Both mouse PanIN and invasive carcinoma were graded using the criteria that are applied to human cancers.

### 2.9 RNA sequencing and library preparation

Fast Blue‐positive DRG were manually collected using a fire‐polished pipette with a diameter of ~25 μm pulled from borosilicate glass capillaries and filled with 1 μL of PBS. Each cell was lysed in 19 μL of 0.4% Triton + 1 μL RNAse inhibitor 40 U·uL^−1^ (Takara, 2313A). Then, 2.4 μL of the lysate was mixed with 1 μL of dNTPs 10 mm (Kappa KK1017) and 1 μL of oligo dT 5′–AAGCAGTGGTATCAACGCAGAGTACT30VN‐3′ 5 μm (Sigma‐Aldrich).

Samples were immediately frozen in liquid Nitrogen and thereafter, the tubes were stored at −80 °C until further processing. scRNA seq on picked DRG was performed at EMBL's Genomic Core Facility, Heidelberg, Germany. Briefly, a modified smart‐seq2 protocol [23] using SuperScript IV RT and tagmentation procedure previously described [24] was used to prepare full‐length cDNA sequencing libraries. Reverse transcription was performed at 52 °C for 15 min followed by enzyme inactivation at 80 °C for 10 min, and cDNA was amplified using 22 PCR cycles. cDNA cleanup and tagmentation were performed using SPRI bead–based purification, and libraries were normalized prior to sequencing.

### 2.10 Nested PCR on sequenced DRG neurons

To amplify low‐abundance transcripts in sequenced DRG, a nested PCR approach targeting full‐length *Pdx1* mRNA was used. cDNA was synthesized from the lysates of 13 individual neurons. For the first PCR reaction, 1 μL of cDNA was amplified using Primer Pair 1 in a standard PCR master mix for 20 cycles (95 °C for 30 s, 60 °C for 10 s, 72 °C for 15 s), following an initial denaturation at 95 °C for 3 min. This first amplification generated a 207 bp product. A second round of amplification was conducted using Primer Pair 2 in a real‐time PCR reaction to produce a 93 bp nested amplicon. The qPCR was performed for 40 cycles (95 °C for 10 s, 60 °C for 10 s) following an initial denaturation at 95 °C for 30 s. The final run included a melting curve analysis with 0.5 °C increments per cycle and 15 s per step. Primer design was based on the mouse *Pdx1* reference sequence NM_008814.4 (Table S1).

### 2.11 RT‐qPCR on sequenced DRG neurons and isolated EVs

RT‐qPCR was performed to validate scRNA‐seq data using cDNA from pooled DRG neurons (4 KPC and 13 WT mice). Lysates from 20 randomly picked cells per animal were combined, diluted, and processed for cDNA synthesis using the Takara kit (RR047A) following the manufacturer's instructions with minor modifications, including genomic DNA digestion and the use of both oligo(dT) primers and random hexamers. The qPCR was performed for 40 cycles (95 °C for 10 s and 60 °C for 10 s per cycle following an initial denaturation step) followed by melting curve analysis with 0.5 °C temperature increments and 15 s per step. EV‐derived RNA was processed using the same protocol, and primers were designed based on transcript mapping (Table S1).

### 2.12 Extracellular vesicle isolation

Extracellular vesicles (EVs) were isolated from three distinct sources: organoids, acute pancreatic tissue slices, and blood plasma of KPC and sibling mice lacking the KRAS mutation but treated with tamoxifen. Organoids were cultured following established protocols [21], and EVs were collected from conditioned media after sequential centrifugation steps to remove debris and large vesicles, followed by ultracentrifugation at high speed to pellet EVs. Acute pancreatic slices were prepared using a McIlwain tissue chopper [25] (Brinkman, Westbury, NY; 350‐μm thick) and maintained on membrane inserts under standard culture conditions before EV harvesting from the culture medium. For blood‐derived EVs, plasma was isolated from whole blood by serial centrifugation steps to eliminate cells, debris, and larger particles, followed by ultracentrifugation to collect EVs. In all cases, the final EV pellet was resuspended in a lysis buffer containing Triton X‐100 and RNaseOUT for downstream analyses.

### 2.13 Mapping and quantification of genes in sequenced cells

Quality control was performed on all FASTQ files received from the sequencing facility using QoRTs [26]. The read files were mapped to the GRCm39 version of the genome assembly using the STAR aligner [27]. First, the *Mus_musculus.GRCm39.dna.primary_assembly.fa* and *Mus_musculus.GRCm39.108.chr.gtf* files were downloaded from Ensembl [28] and used to generate a reference index. This was followed by aligning the reads of each cell to the reference genome one at a time. Finally, gene counts were extracted for each cell using the htseq‐count [29] function, resulting in a gene count matrix for all the sequenced cells.

### 2.14 From bulk sample processing to analyzing single cells using Seurat

Seurat [30], an R package designed for quality control, analysis, and exploration of single‐cell RNA‐seq data, was used for further downstream analyses. First, a Seurat object was created using the CreateSeuratObject() function, by passing the gene count matrix and the quality control metrics calculated for each cell using QoRTs results as metadata. The PercentageFeatureSet() function was used to compute the percentage of counts attributed to mitochondrial and ribosomal genes for each cell. To visualize the quality control parameters, the VlnPlot() function was used. Cells having non‐zero counts for more than 1000 genes were selected for further analysis.

### 2.15 Annotation of cells using Monocle3

The Monocle3 package was used to identify the cell types present in our dataset. A cell dataset object was created for the reference [14] and the query (our data) data, using the new_cell_data_set() function. Both datasets were subsetted to include only common genes. Size factors were re‐calculated using the estimate_size_factors() function. The reference dataset was transformed to low dimensional PCA and UMAP spaces using preprocess_cds() and reduce_dimension() functions. The build_nn_index parameter was set to TRUE to build a nearest neighbor index in the UMAP space, which was later used to transfer the reference annotations to the query. The transformed models and nearest neighbor index were saved using the save_transform_models() function and later loaded into the query dataset with the load_transform_models() function. The query data was projected into the reference space using the preprocess_transform() and reduce_dimension_transform() functions. The two datasets were combined using the combine_cds() function to visualize the overlap of query cells on reference space using the plot_cells() function. Finally, cell type annotations from the reference are transferred to the query dataset using the transfer_cell_labels() and fix_missing_cell_labels() functions. The transferred annotations were added to the metadata of the Seurat object. Cells with unsolved, not‐a‐neuron, and other outlier annotations were excluded from further analysis. The Seurat object was normalized for each mouse using the SCTransform() function. Dimensionality reduction to PCA and UMAP spaces was done using the RunPCA() and RunUMAP() functions with default parameters. Lastly, nearest‐neighbor graph construction and unsupervised clustering (using the default Louvain algorithm) were performed with FindNeighbors() and FindClusters() functions. TheDimPlot() function was used to visualize cells, and the group.by parameter was varied depending on what was to be visualized.

### 2.16 Identification of markers and differential gene expression analysis

The FindAllMarkers() function was used to identify markers of the different DRG populations in the dataset. Genes that are expressed in at least 10% of the cells were tested to return both upregulated and downregulated putative markers of each DRG population. Genes with adjusted *P*‐value (*P*_val_adj) ≤ 0.05 were considered as markers. The DoHeatMap() function was used to plot the heatmap of the top n marker genes of the different DRG populations.

To identify differentially expressed genes between KPC and WT cells, the FindMarkers() function was used, testing genes expressed in at least 10% of total cells. The Wilcoxon Rank Sum test was applied as the statistical test used to identify DEGs in both functions. Genes with *P*_val_adj ≤ 0.05 and absolute log_2_ fold change (abs(avg_log_2_FC)) ≥ 0.25 were considered as differentially expressed (Table S2). The resulting DEGs were visualized with a volcano plot, using the EnhanchedVolcano R package [31].

Gene ontology (GO) analysis was performed for up and downregulated DEGs separately. Genes used for testing in differential expression analysis served as universe for GO analysis. biomaRt R package [32] was used to retrieve gene information (e.g., entrez ID) required for GO analysis. GOstats [33] and GO.db [34] R packages were used to identify the enriched terms for up‐ and downregulated genes, and the results were visualized using ggplot2 R package [35]. The following nomenclature was employed to present results: ns, not significant, *P < 0.05, **P < 0.01, ***P < 0.001, ****P < 0.0001.

## 3 Results

### 3.1 Single‐cell RNA sequencing identifies sensory neuron subtypes innervating healthy pancreatic tissue and PDAC tissue

**Fig. 1:**
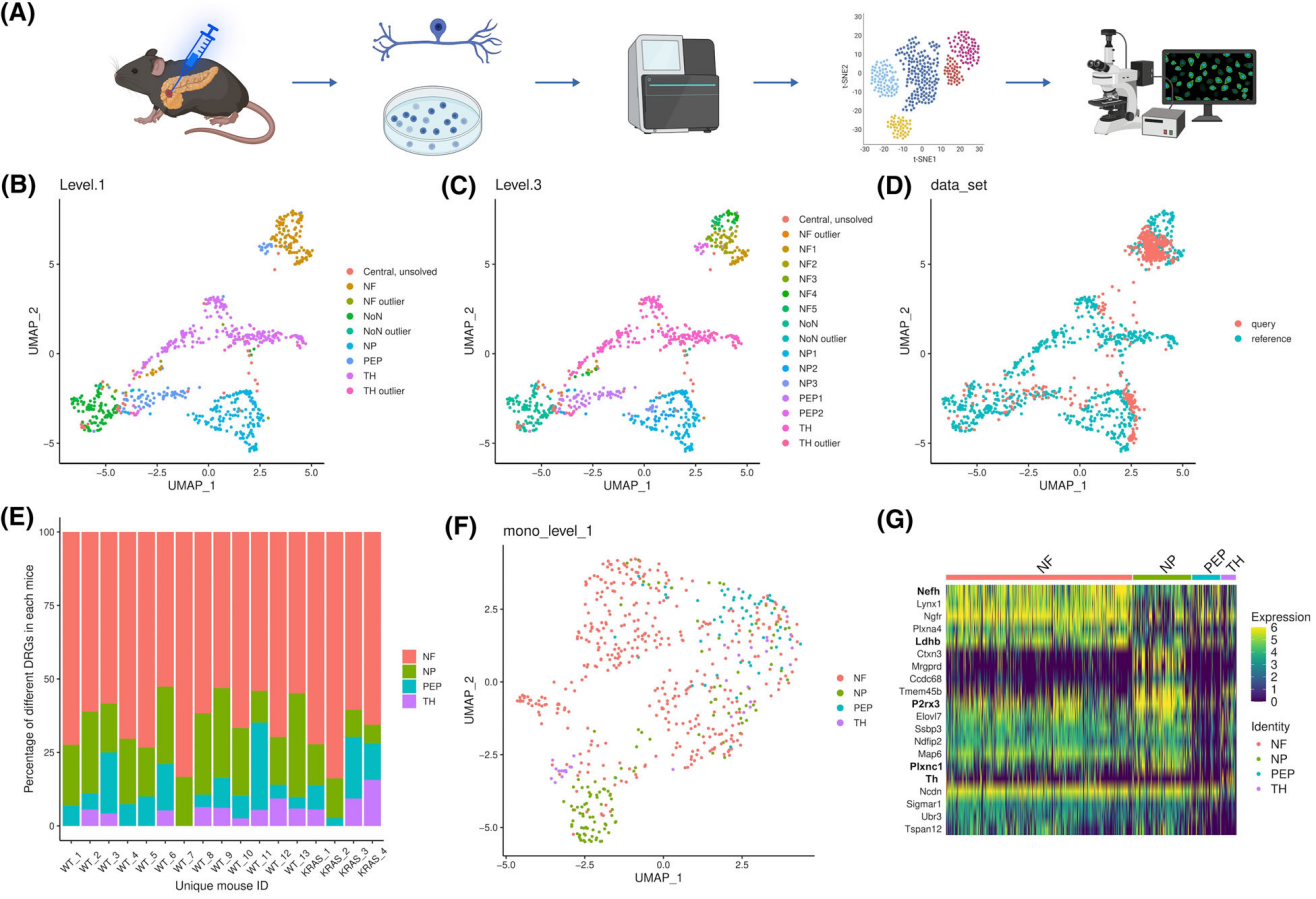
(A) Schematic workflow of the experiment starting from FB injection into healthy or cancer pancreatic tissue, picking of single positive cells after DRG culture, scRNA sequencing, and data analysis. (B, C) Unsupervised clustering of the Usoskin's dataset, cells were visualized by their types and subtypes as assigned in the reference study (NF, neurofilament‐containing; NoN, non‐neuronal cells; NP, non‐peptidergic; PEP, peptidergic; TH, tyrosine hydroxylase. (D) Overlap of our dataset with the reference dataset. E: Distribution of DRG into classes in each mouse. (F) UMAP obtained after Seurat‐based clustering; the color is based on cell assignment done using monocle 3. (G) Heatmap of top 5 marker genes identified after annotation. Highlighted are known marker genes of DRG subtypes that are up‐/downregulated in subtype populations.

To characterize DRG neurons innervating the pancreas, we injected retrograde tracer Fast Blue (FB) into the head and tail regions of the pancreas in 13 wildtype (C57BL/6J) and 4 KPC (Kras^LSL‐G12D^; p53^LoxP^; Pdx1‐CreER) mice. Lumbar and thoracic DRG were dissociated, and FB+ cells were hand‐picked and processed for sequencing. A total of 715 cells were sequenced and analyzed (Fig. 1A). Evaluation of pancreatic tissue sections confirmed preinvasive PanIN or PDAC lesions in KPC mice (Fig. S1).

Quality control parameters were calculated for each cell, including the number of genes identified, the number of RNA molecules detected, and the total ribosomal and mitochondrial content (Fig. S2A). Cells that had non‐zero counts for fewer than 1000 genes were removed from the subsequent analysis. A total of 620 cells remained for further analysis, with an average of 35 cells per animal (Fig. S2B). Unsupervised clustering using Seurat with default parameters identified six discrete clusters (Fig. S2C). However, the top markers of each cluster (Fig. S2D) did not represent discrete DRG subtypes.

To more reliably identify DRG subtypes, we used the validated sensory neuron dataset from Usoskin *et al*. (GSE59739) as a reference. Upon reanalyzing the Usoskin data, we observed that cells clustered according to the original annotations assigned in the study (Fig. 1B,C). Guided by Monocle3 pipelines (methods), we projected our data onto the Usoskin reference map (Fig. 1D). Most of the cells clustered within the NF (neurofilament‐containing) subtype, while fewer cells mapped to NP (non‐peptidergic), PEP (peptidergic), or TH (tyrosine hydroxylase–expressing) subtypes. Cells that were annotated as outliers, non‐neuronal, or lacked annotations were excluded from further analysis, leaving behind 603 cells. By examining annotated DRG subtype proportions across individual mice, we consistently observed a dominance of NF neurons and fewer TH cells in both wild‐type and KPC cohorts (Fig. 1E). Notably, the Seurat‐based clustering approach also showed concordance with these annotated subtypes (Fig. 1F). As an additional validation, we extracted top marker genes for each annotated DRG subtype and compared them to known DRG markers (Fig. 1G). The NF population showed high expression of *Nefh* and *Ldhb*, whereas *Plxnc1* and *P2rx3* were elevated in NP neurons; notably, *Plxnc1* was downregulated in PEP cells, and *Th* was enriched in the TH neurons. These expression patterns align with established DRG subtype‐specific markers, confirming the robustness of the reference‐based annotation and providing a solid framework to proceed with further exploration of DRG roles in pancreatic function and the progression of PDAC.

### 3.2 NF neurons predominate in pancreatic innervation, while NP neurons decline in PDAC

**Fig. 2:**
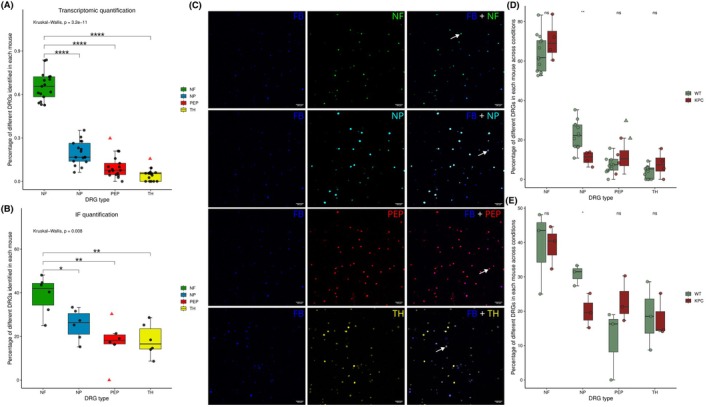
(A) Transcriptomic quantification of Fast Blue–positive DRG neurons across neuronal classes (NF, NP, PEP, TH) (Kruskal–Wallis test, *P* = 3.2e‐11). (B) Immunofluorescence (IF) quantification of Fast Blue–positive DRG neurons across neuronal classes (Kruskal–Wallis test, *P* = 0.008). (C) Immunostaining of Fast Blue‐positive DRG with antibodies against Neurofilament 200 (NF), Isolectin B4 (NP), Substance P (PEP), Tyrosine Hydroxylase (TH). Arrows indicate representative examples of colocalization between Fast Blue positive labeling and the indicated marker and are provided for illustrative purposes only. Scalebar = 100 μm. (D) Transcriptomic quantification of Fast Blue–positive DRG neurons across neuronal classes, stratified by genotype (KPC vs WT) (*n* = 4 KPC; *n* = 13 WT; *t*‐test, *P* = 0.0013). (E) Immunofluorescence quantification of Fast Blue–positive DRG neurons across neuronal classes, stratified by genotype (*n* = 3 KPC; *n* = 3 WT; *t*‐test, *P* = 0.044). Boxplots show the median (center line), interquartile range (box), and whiskers extending to 1.5 × IQR; individual points represent biological replicates.

The quantification of DRG subtypes using RNAseq across all 17 mice revealed a consistent abundance of NF neurons, comprising an average of 65% of the total DRG identified per mouse. NP neurons were the second most frequent subtype, with an average of 20%, followed by PEP and TH neurons, which accounted for 10% and 5%, respectively (Fig. 2A). The predominance of NF neurons was evident across both KPC and WT mice and was statistically significant (Kruskal–Wallis test, *P* < 0.01).

To further validate and substantiate our transcriptomic findings, we performed immunofluorescence (IF) on cultured DRG from 6 mice (3 KPC and 3 WT). We used anti‐NF200, anti‐Substance P, Isolectin B4, and anti‐TH antibodies to identify NF, PEP, NP, and TH neurons, respectively. The IF results (Fig. 2B,C) corroborated our single‐cell observations, revealing specific fractions of NF, NP, PEP, and TH DRG innervating the pancreas. Again, NF neurons emerged as the most abundant DRG subtype, comprising an average of 39% of total FB+ cells, followed by NP neurons at 25%. PEP and TH neurons accounted for roughly 18% each. The enrichment of NF neurons was statistically significant (Kruskal–Wallis test, *P* < 0.01) and remained consistently high in both KPC and WT mice in terms of relative abundance (Fig. 2B).

The transcriptomic data revealed a significant reduction in the proportion of NP sensory neurons in KPC mice compared to WT mice (average NP KPC 10.73% vs NP WT 22.36% *t*‐test, *P*‐value 0.0013) (Fig. 2D). No significant differences were found for the other DRG subtypes between the conditions. The IF analysis confirmed the significant reduction in the proportion of NP neurons in KPC mice (Fig. 2E and Fig. S3A): FB+ NP cells in KPC was 20% versus 30.7% in WT, *t*‐test, *P* = 0.044. We also performed further immunofluorescence analysis of FastBlue‐negative cells, and we observed no difference in the proportion of any neuronal population in both WT and KPC mice (Fig. S3B), indicating that there is no preference in retrotracer uptake by specific Fast Blue‐positive populations.

Motivated by these findings, we sought to perform immunostaining in pancreatic slices to understand whether distinct neuronal subpopulations could also be detected in the pancreas of WT and KPC mice. While we were able to localize axons positive for the markers NF200 and TH (corresponding to the “NF” and “TH” neuronal populations, respectively), we did not observe a clear predominance of one subtype over the other in WT or KPC mice (Fig. S4A,C). We could not detect any axons positive for IB4 (corresponding to “NP” DRG neurons), likely due to the low expression of α‐galactose residues in distal axons of this neuronal class, which IB4 binds to (Fig. S4B). Because the size and heterogeneity of the pancreas in the KPC model preclude robust and unbiased quantification of NF200‐positive fibers, pancreatic IF was used exclusively for qualitative illustration, and quantitative analyses were restricted to the dorsal root ganglia.

### 3.3 Novel detection of *Pdx1‐CreERT2* and upregulation of *Snca* in KPC DRG suggest evidence of EV‐mediated transfer

**Fig. 3:**
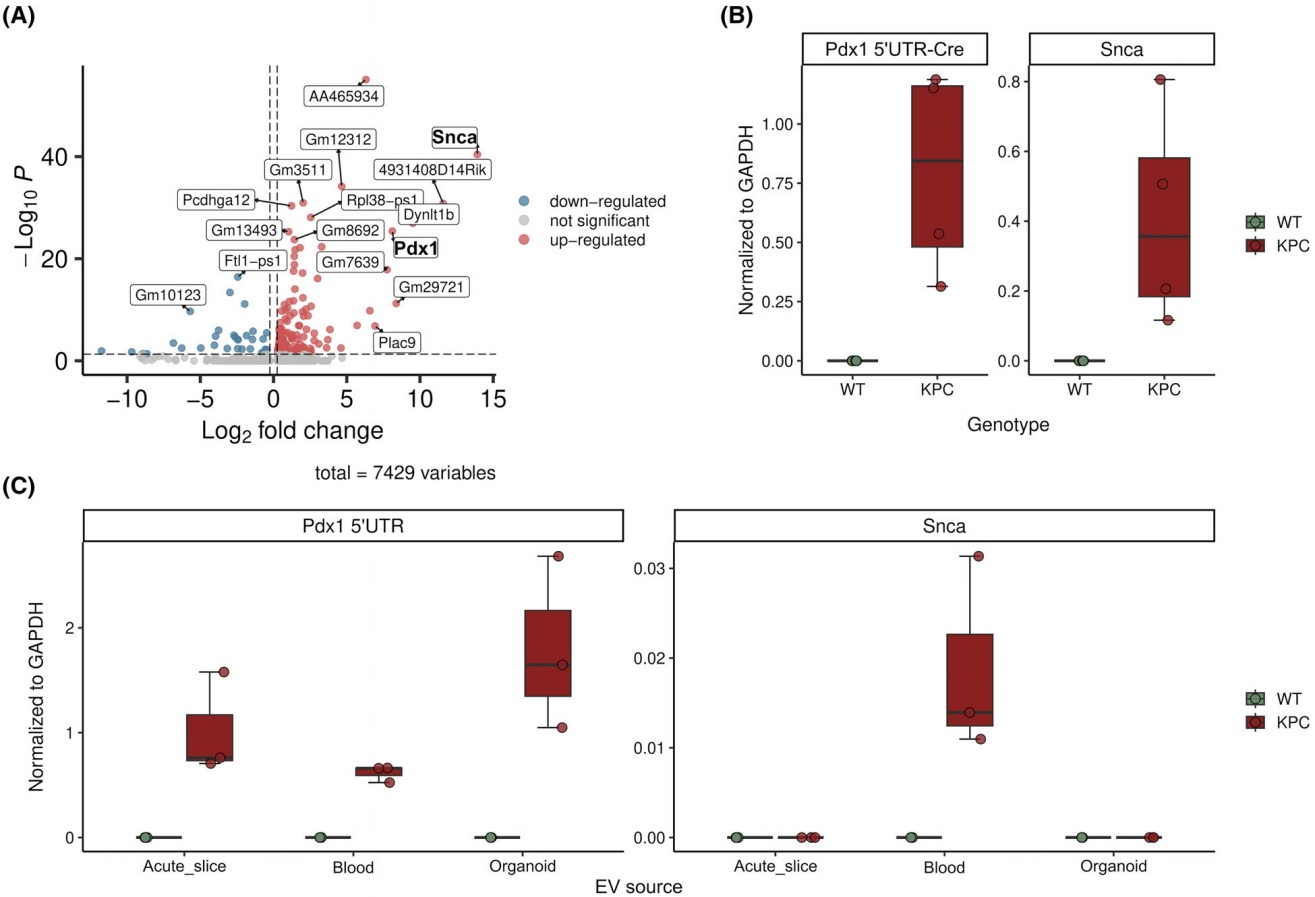
(A) Volcano plot highlighting *Pdx1* 5′UTR and *Snca* among the most upregulated genes (red), and immune system‐related genes among the downregulated (blue) (total genes tested = 7429) (B) RT‐qPCR validation of Pdx1 5′UTR and Snca expression across genotypes (*n* = 4 KPC; *n* = 13 WT). Twenty cells were pooled per animal prior to RNA extraction. Expression was normalized to GAPDH and to gene‐specific positive controls. For Snca, the positive control consisted of 10 ng cDNA from 10^6^ cortical neurons (C57BL/6J WT). For Pdx1 5′UTR, the positive control consisted of 10 ng total DNA extracted from the pancreas of a tamoxifen‐treated KPC mouse. (C) RT‐qPCR validation of Pdx1 5′UTR and Snca in extracellular vesicles (EVs) derived from organoids, acute slices, and blood (*N* = 3 per group). KPC: Kras^*LSL‐G12D+/+; p53LoxP; Pdx1‐CreER*^ mice. WT: Kras^*LSL‐G12D−/−; p53LoxP; Pdx1‐CreER*^ siblings. All mice were treated with tamoxifen. No statistical test was applied, as transcripts were not detected in WT samples. Boxplots show the median (center line), interquartile range (box), and whiskers extending to 1.5 × IQR; individual points represent biological replicates.

Differential expression analysis between KPC and wild‐type (WT) DRG neurons revealed 180 significantly altered genes, including 149 upregulated and 31 downregulated in KPC DRG (Fig. 3A, Table S2). Among the upregulated genes, *Pdx1* (*adj. P* = 3.92 × 10^−26^) and *Snca* (*adj. P* = 3.83 × 10^−41^) were particularly interesting as they were detected exclusively in KPC neurons (*Pdx1* ^+^ − 31%; *Snca* ^+^ − 40%, of all KPC DRG) with most positive cells falling within the NF subtype (Fig. S5). Further inspection showed that *Pdx1* reads were predominantly mapped to the 5′UTR region of the gene (Fig. S6). Consistent with the transcriptomic findings, RT‐qPCR using 5′UTR–specific primers for *Pdx1* (Table S1) confirmed the exclusive expression of the transcript in KPC DRG, whereas WT DRG were devoid of these signals (Fig. 3B, Figs S7 and S8A) as well as DRG from control KPC mice not treated with tamoxifen (Fig. S8B, left). Intriguingly, we did not detect canonical *Pdx1* transcript in any of these samples (the functionality of both Pdx1 full‐length primer pairs 1 and 2 was confirmed in cDNA extracted from normal pancreas organoids, Fig. S8B, right).

We reasoned that the 5′UTR‐truncated *Pdx1* transcripts in KPC DRG may be derived from the exogenous CreERT2 transgene that is preceded by a portion of the *Pdx1* promoter and integrated randomly into the genome of KPC [36]. We thus re‐examined the sequencing data for the presence of either this transgene or the endogenous *Pdx1* transcript in those DRG positive for *Pdx1* 5′UTR reads. As shown in Fig. S9, overlap with CreERT2 sequence was evident in all samples, while endogenous *Pdx1* reads extending beyond the 5′UTR were never detected. This was experimentally validated using RT‐PCR to generate amplicons that extended from the 5′UTR into either the CreERT2 transgene or endogenous canonical *Pdx1*. Again, CreERT2 amplicons were detected at similar levels to the 5′UTR transcripts in DRG from KPC mice (Fig. S10), while endogenous canonical *Pdx1* transcripts were never observed. We further analyzed published RNAseq datasets from 3 different studies (Usoskin [14]; Zeisel [17] and Sharma [37]) for the presence of the *Pdx1* transcript. In agreement with our own data, we confirmed that the *Pdx1* transcript was not present in any population of DRG neurons from wildtype animals, further supporting our hypothesis that the 5′UTR of the CreERT2 transgene is detected in the DRG only in KPC animals.

Given these unexpected findings, we hypothesized that the presence of *Pdx1‐Cre* and *Snca* transcripts in DRG might originate from circulating EVs. To test this, we isolated EVs from three complementary models—(1) *in vitro* (organoids), (2) *ex vivo* (acute slices), and (3) *in vivo* (blood) samples—and probed for 5′ UTR *Pdx1‐Cre* and *Snca* by RT‐qPCR. Strikingly, *Pdx1* 5′UTR was consistently detected in vesicles from all three KPC models, including blood‐derived EVs, but was absent from WT controls (Fig. 3C; Fig. S11). Conversely, *Snca* was detected only in EVs derived from KPC blood, remaining undetectable in KPC organoid– or acute slice–derived EVs and in all WT EV samples (Fig. 3C; Fig. S11). Collectively, the sequencing data from DRG neurons and the corresponding RT‐qPCR experiments validate the unique presence of *Pdx1‐Cre* and *Snca* transcripts in KPC DRG, and further analysis of these transcripts in vesicles suggests that they may be selectively packaged and transferred via circulating EVs *in vitro* and *in vivo*.

### 3.4 Functional enrichment analysis reveals altered mitochondrial activity in KPC DRG

**Fig. 4:**
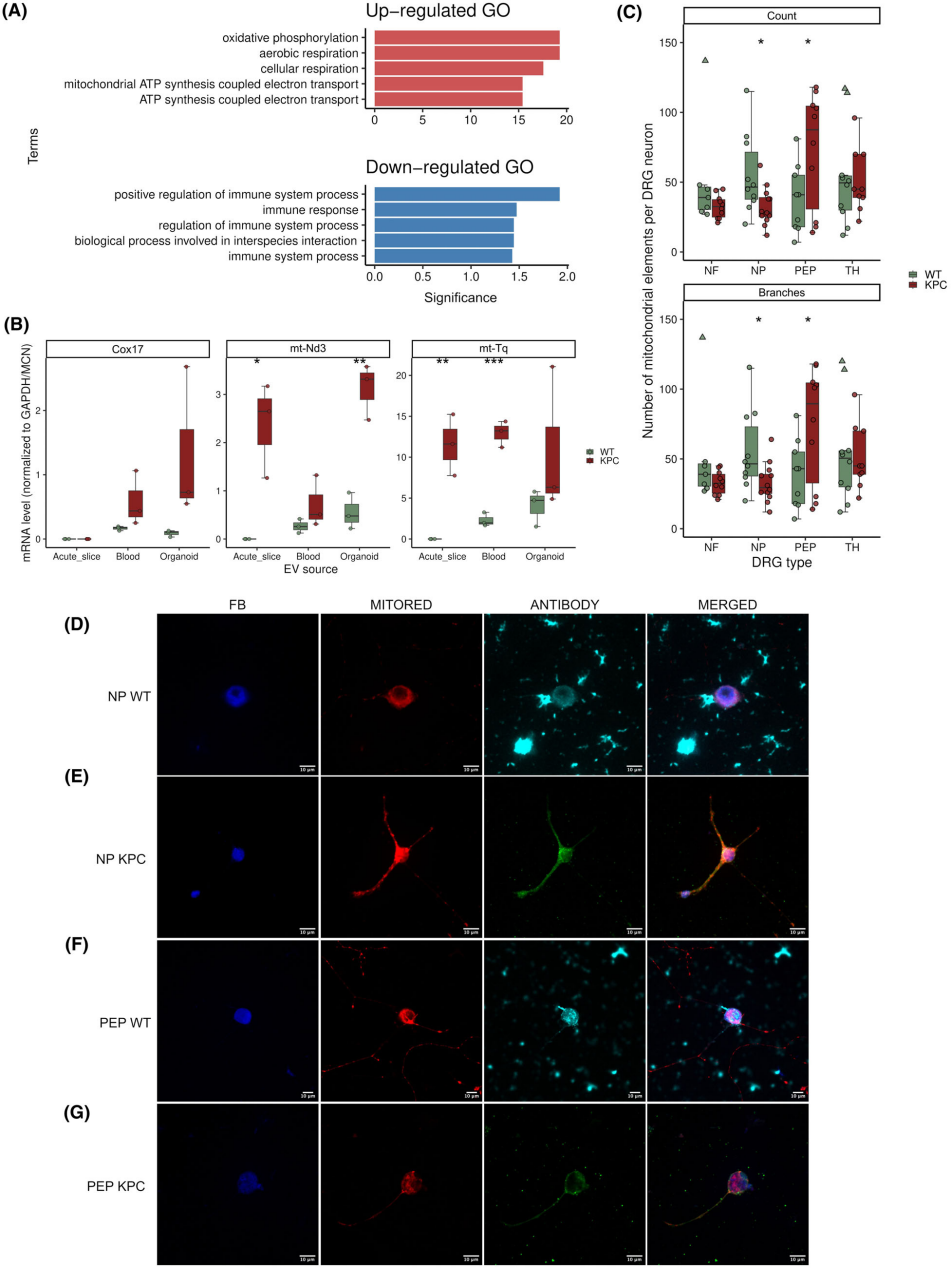
(A) Gene ontology (GO) analysis of Fast Blue–positive DRG neurons comparing KPC and WT mice. Red bars indicate upregulated pathways; blue bars indicate downregulated pathways. (B) mRNA expression levels of *Cox17*, *mt‐ND3*, and *mt‐tQ* in extracellular vesicles (EVs) derived from organoids, acute slices, and blood (*n* = 3 WT; *n* = 3 KPC). Expression levels were normalized to GAPDH and to a gene‐specific positive control (10 ng cDNA from pancreatic tissue of a tamoxifen‐treated KPC mouse). Statistical analysis was performed using two‐way ANOVA followed by *post hoc* pairwise comparisons (WT vs KPC within each EV source). KPC: *Kras* ^*LSL‐G12D+/+; p53LoxP; Pdx1‐CreER*^ mice. WT: *Kras* ^*LSL‐G12D−/−; p53LoxP; Pdx1‐CreER*^ siblings. All mice were treated with tamoxifen. (C) Quantification of mitochondrial number and branching in DRG neurons (*n* = 10 neurons per group, derived from 1 WT and 1 KPC animal). Statistical analysis was performed using two‐way ANOVA followed by *post hoc* pairwise comparisons within each DRG subtype (Count NP *P*.adj = 0.0259, PEP *P*.adj = 0.0437; Branches NP *P*.adj = 0.0282, PEP *P*.adj = 0.044). (D–G) Representative images of Mitored and immunostaining for NP (Isolectin B4) and PEP (Substance P) in WT and KPC Fast Blue–positive DRG neurons. Boxplots show the median (center line), interquartile range (box), and whiskers extending to 1.5 × IQR; individual points represent biological replicates or DRG neurons.

To gain insight into the functional pathways disrupted in KPC DRG, we performed gene ontology (GO) enrichment analysis on the sets of differentially expressed genes. Notably, upregulated genes were enriched for mitochondrial processes, whereas downregulated genes were linked to immune system processes (Fig. 4A). Motivated by these findings and our earlier observations of EVs transfer, we sought to validate three mitochondrial‐related genes—*mt‐ND3, Cox17*, and *mt‐tQ*—previously implicated in cancer and mitochondrial pathways [38, 39, 40]. Using RT‐qPCR on EVs isolated from the three models (*in vitro* organoids, *ex vivo* acute slices, and *in vivo* blood), we observed that these genes were largely enriched in KPC EVs (Fig. 4B) (mt‐ND3 EVs KPC vs. WT, *P* < 0.05; mt‐Tq EVs KPC vs WT, *P* < 0.01, two‐way ANOVA, followed by *post hoc* pairwise *t*‐tests). In contrast, when we examined pooled DRG (Fig. S12), these mitochondrial transcripts did not show similar enrichment. We attribute this discrepancy to the random selection of DRG for pooling in each mouse, which may have masked differences in the expression of these genes due to subtype‐specific variations in total mitochondrial content between WT and KPC DRG.

To further explore potential mitochondrial alterations, we performed mitochondrial staining with MitoRed on FB^+^ DRG from WT and KPC mice. While no significant differences were evident at the overall population level (Fig. S13), subtyping revealed marked variations (Fig. 4C). Specifically, KPC PEP neurons displayed both higher mitochondrial counts and more extensive branching compared with WT PEP neurons (two‐way ANOVA, *post hoc* pairwise *t*‐tests within each DRG type, *P* < 0.05), whereas KPC NP neurons exhibited lower mitochondrial content and fewer branches compared with WT NP neurons (two‐way ANOVA, *post hoc* pairwise *t*‐tests within each DRG type, *P* < 0.05). Representative images of FB^+^ DRG (Fig. 4D–G) illustrate these subtype‐specific differences, underscoring the heterogeneous mitochondrial responses within distinct DRG populations.

## 4 Discussion

Here, we used single‐cell RNA sequencing of retrogradely labeled DRG neurons to profile sensory subtypes, innervating healthy pancreas, PanIN, and early PDAC, and to identify disease‐associated transcriptional changes.

Because initial unsupervised clustering did not cleanly resolve known DRG classes, we anchored our dataset to the well‐characterized DRG dataset generated by Usoskin and colleagues, who utilized comparable methodologies for single‐cell profiling [14]. We selected Usoskin's study as reference dataset over other available resources for several reasons: The study by Zeisel *et al*. [15] surveyed the entire mouse nervous system, lacking a dedicated emphasis on sensory neurons; the dataset from Hockley *et al*. [17] covered only thoracolumbar and lumbosacral DRG rather than the full range of DRG populations; meanwhile, the work by Kupari *et al*. [16] was based on primate rather than mouse DRG. Notably, both Hockley et al. and Kupari et al. also referenced Usoskin *et al*.'s classification framework [14]. Guided by this reference, we classified our DRG into four canonical sensory neuron subtypes: neurofilament‐containing (NF), non‐peptidergic (NP), peptidergic (PEP), and tyrosine hydroxylase–expressing (TH) neurons. Consequently, it serves as a foundation for further exploration of the molecular mechanisms underpinning neuronal involvement in early PDAC progression. Notably, the numerical imbalance between WT and KPC cohorts used for sequence is a result of the intrinsic breeding constraints of the KPC model and the stringent requirement for histological confirmation of PDAC prior to inclusion. This approach, while reducing the final number of KPC animals, ensured that downstream analyses focused exclusively on tumor‐associated sensory neurons. Importantly, retrograde labeling and single‐cell dissociation efficiencies were comparable across groups, and analyses were conducted at the single‐cell level with robust per animal normalization, minimizing the risk of bias due to sample size differences.

A striking and consistent finding was the dominance of NF neurons among pancreatic‐innervating sensory neurons. NF neurons accounted for approximately two‐thirds of retrogradely labeled cells in the scRNA‐seq dataset and ~40% by immunofluorescence, in agreement with recent reports showing NF enrichment in PDAC‐innervating sensory neurons [41].

Although NF neurons are classically associated with proprioception and mechanosensation, their prevalence suggests an underappreciated role in sensing mechanical or tissue perturbations within the pancreas. Given the known sensitivity of pancreatic tissue to mechanical stress and the involvement of Piezo mechanosensitive channels in pancreatic cell types, NF neurons may be well positioned to detect early biomechanical changes associated with tumor development [42, 43].

In contrast to the stability of NF, PEP, and TH neuron proportions, NP neurons were selectively reduced in KPC mice, a finding consistently observed across transcriptomic and histological analyses [41]. Altered sensory innervation is a recognized feature of tumor microenvironments [44, 45], and NP neurons have been implicated in regulating local immune responses [46]. Their loss may therefore contribute to dysregulated neuroimmune signaling and pro‐inflammatory conditions that promote tumor progression, identifying NP neurons as a potential target for early neuroimmune intervention in PDAC. Transcriptomic and immunofluorescence data were highly concordant, supporting the robustness of our conclusions despite modest differences in subtype proportions, likely reflecting intrinsic differences between RNA‐ and protein‐based detection methods rather than technical issues such as retrograde labeling efficiency or sampling bias. Together, these findings establish a clear shift in the composition of pancreatic sensory innervation during early PDAC. Unexpectedly, we detected upregulated DRG transcripts in extracellular vesicle (EV) preparations from multiple sources (the most relevant of which being blood), raising the possibility of EV‐mediated RNA transfer between tumors and neurons [47, 48, 49, 50]. In particular, *Snca* and several mitochondrial genes *Cox17*, *mt‐ND3*, *mt‐tQ*, each associated with tumor cell survival and cancer progression [38, 39, 40] were enriched in plasma‐derived EVs from KPC mice. *Snca* was selectively detected in KPC DRG and plasma‐derived EVs, consistent with reports linking α‐synuclein to PDAC progression and perineural invasion [51]. These findings suggest that EV‐mediated signaling may contribute to neuronal remodeling in early PDAC and highlight the potential of EV cargo as early biomarkers of disease. Although the exact origin of *Snca* in DRG remains to be defined, these findings are consistent with models in which α‐synuclein is packaged and transferred via EVs under cellular stress or lysosomal dysfunction, as demonstrated in other pathological contexts [52, 53]. The detection of Pdx1‐CreERT2 transcripts in EVs and DRG also raises important considerations for Cre‐based cancer models. Although recombination in the inducible KPC model requires tamoxifen, EV‐mediated transfer of Cre transcripts could theoretically contribute to off‐target recombination in constitutive Cre systems, warranting further investigation. Finally, we observed upregulation of mitochondrial and oxidative phosphorylation pathways in KPC DRG. Mitochondrial morphology was also altered, with increased abundance and branching in PEP neurons, in contrast to reduced mitochondrial content and complexity in NP neurons. Given the concurrent reduction in NP neurons and their potential role in immune modulation [44], these metabolic changes further underscore the functional remodeling of tumor‐innervating sensory neurons during early PDAC.

## 5 Conclusions

In summary, our study identifies the sensory neuron subtypes innervating the pancreas and reveals selective remodeling of this innervation during early PDAC. NF neurons constitute the majority of pancreatic sensory innervation, whereas NP neurons are selectively depleted during early PDAC. In parallel, DRG neurons exhibit tumor‐associated transcriptional reprogramming, possibly influenced by extracellular vesicle‐mediated signaling. These findings advance our understanding of neuron‐tumor interactions in pancreatic cancer and suggest new avenues for diagnostic and therapeutic strategies targeting the sensory nervous system.

## Conflict of interest

The authors declare no conflict of interest.

## Author contributions

EG, MM and UR are joint first authors. EG and PH are corresponding authors. Conceptualization: PH. Data curation: EG, MM and UR. Formal analysis: EG and UR. Funding acquisition: PH. Investigation: EG, MM, UR, PH; RS. Methodology: EG, MM, UR, FD, RDF, IS, DF, GD, NZ, MC, LV. Project administration: EG, PH, RS. Resources: PH. Supervision: EG, PH and RS. Writing –original draft: EG, MM and UR. Writing – review and editing: EG, MM, UR, PH, RS, VC, FD, IS, IL, DF, GD, NZ, MC, LV.

## Supporting information


**Fig. S1.** Histological characterization of murine pancreatic tissue (H&E staining).
**Fig. S2.** scRNA‐seq quality control and clustering of DRG neurons.
**Fig. S3.** Retrograde tracing and marker expression in DRG neurons.
**Fig. S4.** Sensory neuron subpopulation axonal localization in pancreatic tissue.
**Fig. S5.** Expression of Pdx1 5′UTR and Snca in WT and KPC DRG neurons.
**Fig. S6.** IGV visualization of Pdx1 5′UTR reads in DRG neurons.
**Fig. S7.** RT‐qPCR validation of Pdx1 5′UTR in pooled DRG neurons.
**Fig. S8.** Agarose gel detection of Pdx1 5′UTR in DRG neurons and controls.
**Fig. S9.** Schematic of scRNA‐seq reads from KPC and WT pancreatic DRG neurons.
**Fig. S10.** RT‐PCR detection of Pdx1 5′UTR and Pdx1 5′UTR‐Tg Cre in DRG neurons.
**Fig. S11.** Pdx1 5′UTR and Snca detection in blood‐derived EVs from KPC and WT mice.
**Fig. S12.** RT‐qPCR validation of mitochondrial genes (mttQ, Cox17, mt‐ND3) in DRG neurons.
**Fig. S13.** Mitochondrial quantification in FB+ DRG neurons from WT and KPC mice.
**Table S1.** Primers used in this study.

**Table S2.** Differentially expressed genes in KPC versus WT DRG neurons (149 upregulated, 31 downregulated).

## Data Availability

The transcriptomic data reported in this paper are available at the Single Cell Portal under accession number SCP3142. The gene level‐normalized expression data can be navigated at: https://singlecell.broadinstitute.org/single_cell/study/SCP3142/pancreatic‐ductal‐adenocarcinoma‐reshapes‐sensory‐innervation‐deciphering‐transcriptional‐plasticity‐through‐sc‐rna‐seq.
