# Supplementary material for: Deciphering transcriptional plasticity in pancreatic ductal adenocarcinoma reveals alterations in sensory neuron innervation
Source: Mol Oncol. 2026 Mar 18;20(7):1726–41. doi: 10.1002/1878-0261.70233 (PMC13352961; doi:10.1002/1878-0261.70233)
Supplement: Supplementary file 1 — Fig. S1. Histological characterization of murine pancreatic tissue (H&E staining). Fig. S2. scRNA‐seq quality control and clustering of DRG neurons. Fig. S3. Retrograde tracing and marker expression in DRG neurons. Fig. S4. Sensory neuron subpopulation axonal localization in pancreatic tissue. Fig. S5. Expression of Pdx1 5′UTR and Snca in WT and KPC DRG neurons. Fig. S6. IGV visualization of Pdx1 5′UTR reads in DRG neurons. Fig. S7. RT‐qPCR validation of Pdx1 5′UTR in pooled DRG neurons. Fig. S8. Agarose gel detection of Pdx1 5′UTR in DRG neurons and controls. Fig. S9. Schematic of scRNA‐seq reads from KPC and WT pancreatic DRG neurons. Fig. S10. RT‐PCR detection of Pdx1 5′UTR and Pdx1 5′UTR‐Tg Cre in DRG neurons. Fig. S11. Pdx1 5′UTR and Snca detection in blood‐derived EVs from KPC and WT mice. Fig. S12. RT‐qPCR validation of mitochondrial genes (mttQ, Cox17, mt‐ND3) in DRG neurons. Fig. S13. Mitochondrial quantification in FB+ DRG neurons from WT and KPC mice. Table S1. Primers used in this study. [file MOL2-20-1726-s002.docx]

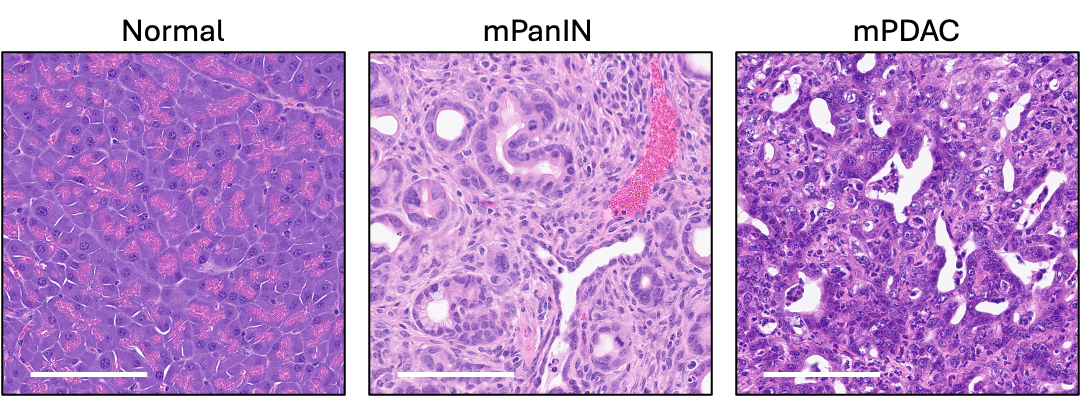


Fig. S1. Hematoxylin and Eosin (H&E) staining of murine pancreatic tissue used for sequencing analysis. From left to right: normal pancreatic tissue from a wild-type C57BL/6j mouse; PanIN ductal structures from a KPC mouse; invasive ductal carcinoma from a KPC mouse. Scale bar, 100 µm.


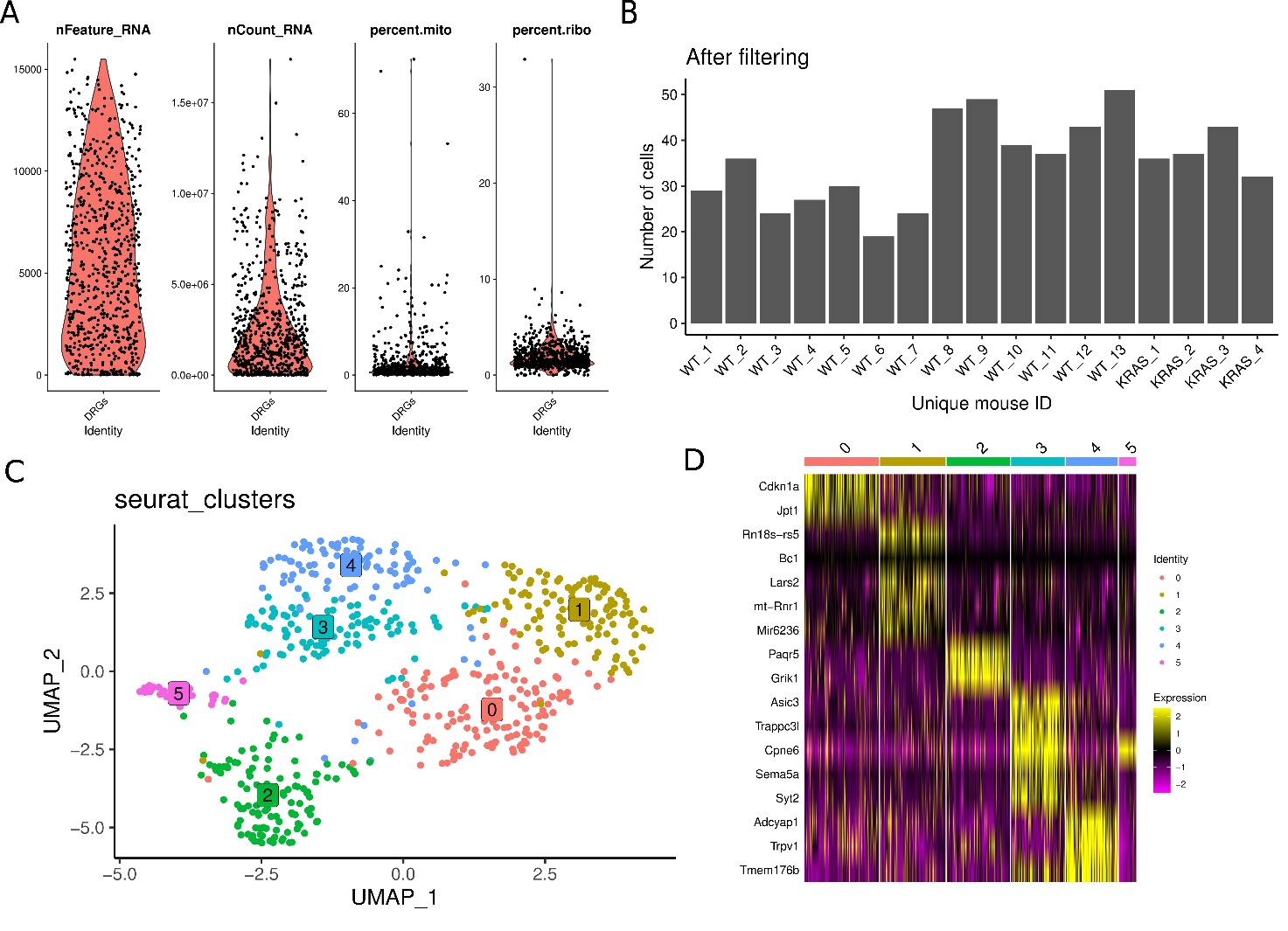


Fig. S2. A: Quality control parameters analysis calculated for each cell, including the number of genes identified, RNA molecules detected, and ribosomal and mitochondrial content. B: Cells with fewer than 1,000 detected genes or high ribosomal/mitochondrial content were excluded, leaving 620 cells for further analysis. C: Identification of 6 unsupervised clusters using Seurat. D: Examination of the top markers revealed that the clustering did not capture discrete DRG types.


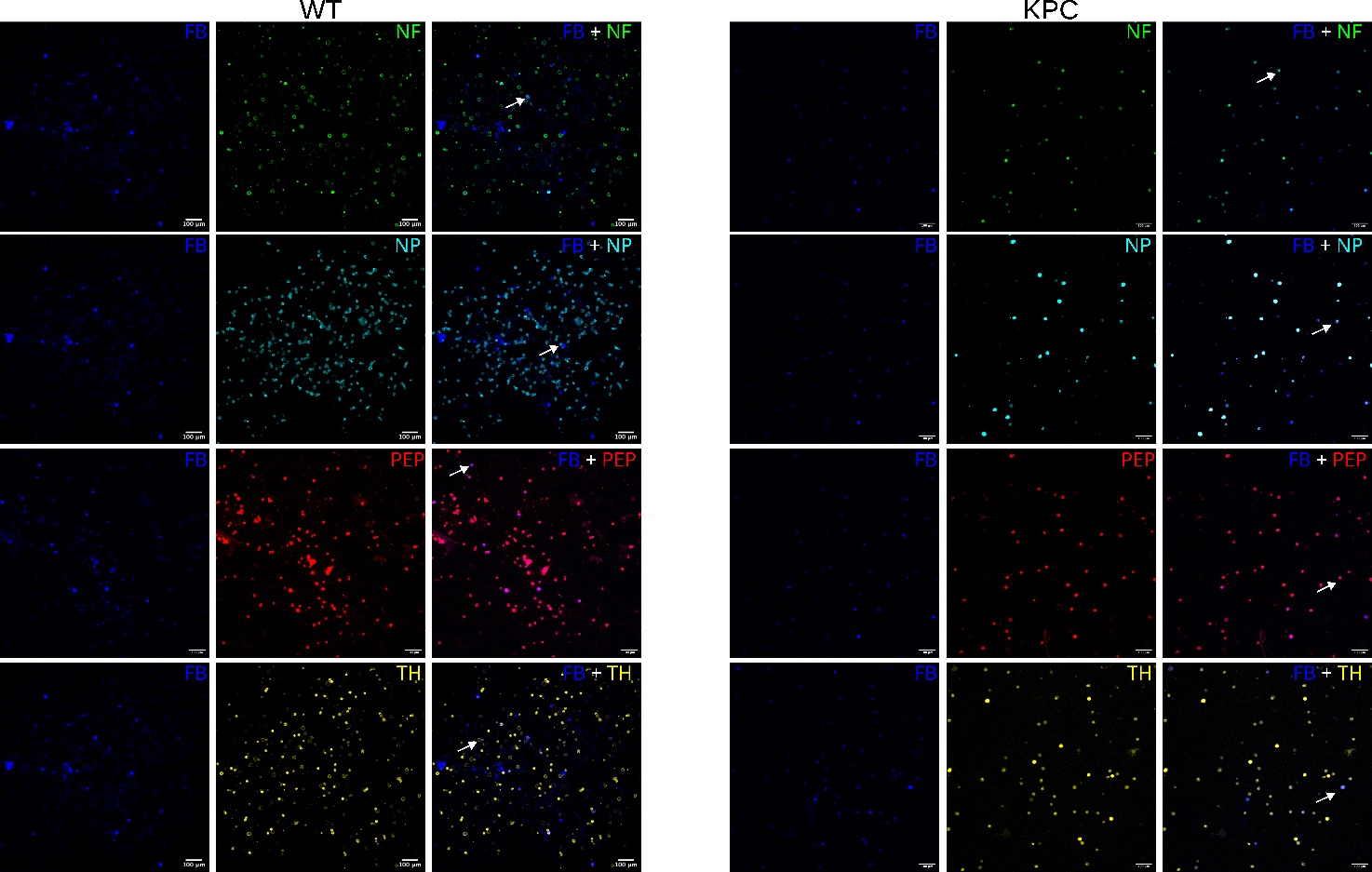


A


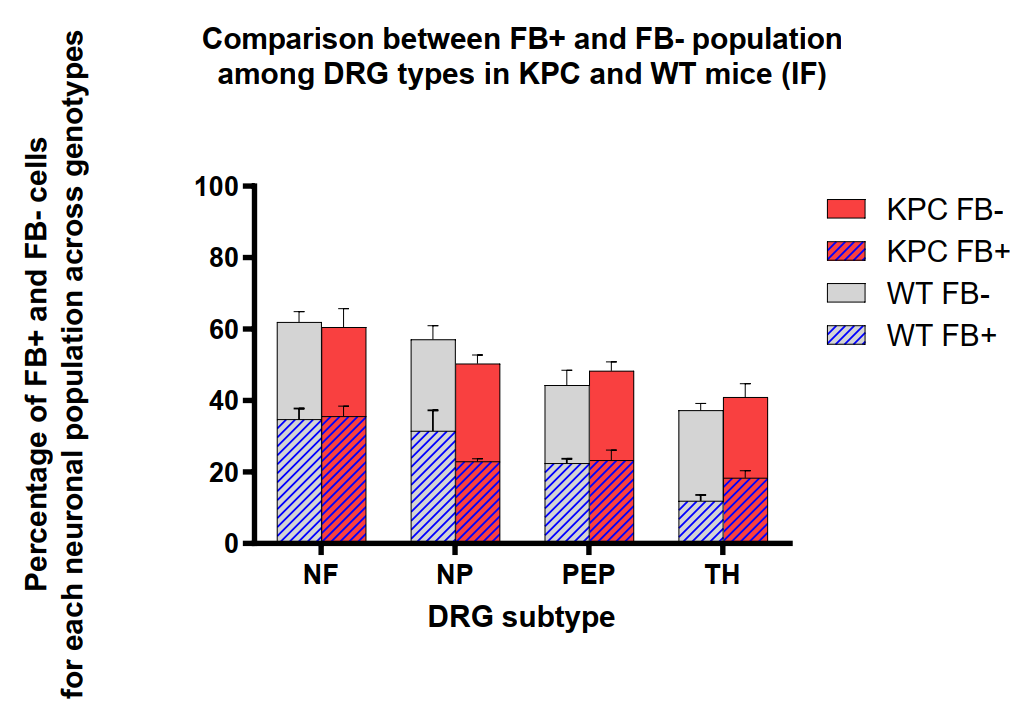


B

Fig S3. A: Immunostaining Fast Blue-positive DRGs from wild-type (WT) or KPC mice using antibodies against Neurofilament 200 (NF), Isolectin B4 (IB4), Substance P (SP), and Tyrosine Hydroxylase (TH). Arrows indicate representative examples of colocalization between Fast Blue positive labeling and the indicated markers are provided for illustrative purposes only (N KPC=3; N WT=3), scalebar=100 µm. B: Immunostaining quantification of FastBue-negative (FB-) and FastBlue positive (FB+) DRG identified in each mouse for each neuronal population, indicating that there is no preference in retrotracer uptake by specific FB+ populations. Images were taken within fields of view where FB+ cells predominated, thus these numbers do not reflect the absolute proportions of FB+ versus FB- cells. Mean of % FB- cells in WT vs KPC (N WT=3; N KPC=3): NF 27.2 vs 25; NP 25.6 vs 27.3; PEP 21.9 vs 25; TH 25.3 vs 22.7. Mean of % FB+ cells in WT vs KPC (N WT=3; N KPC=3): NF 36.6 vs 35.6; NP 29.9 vs 22.8; PEP 20.3 vs 23.3; TH 13.3 vs 18.4; (N KPC=3; N WT=3). Error bars represent the mean ± SD.


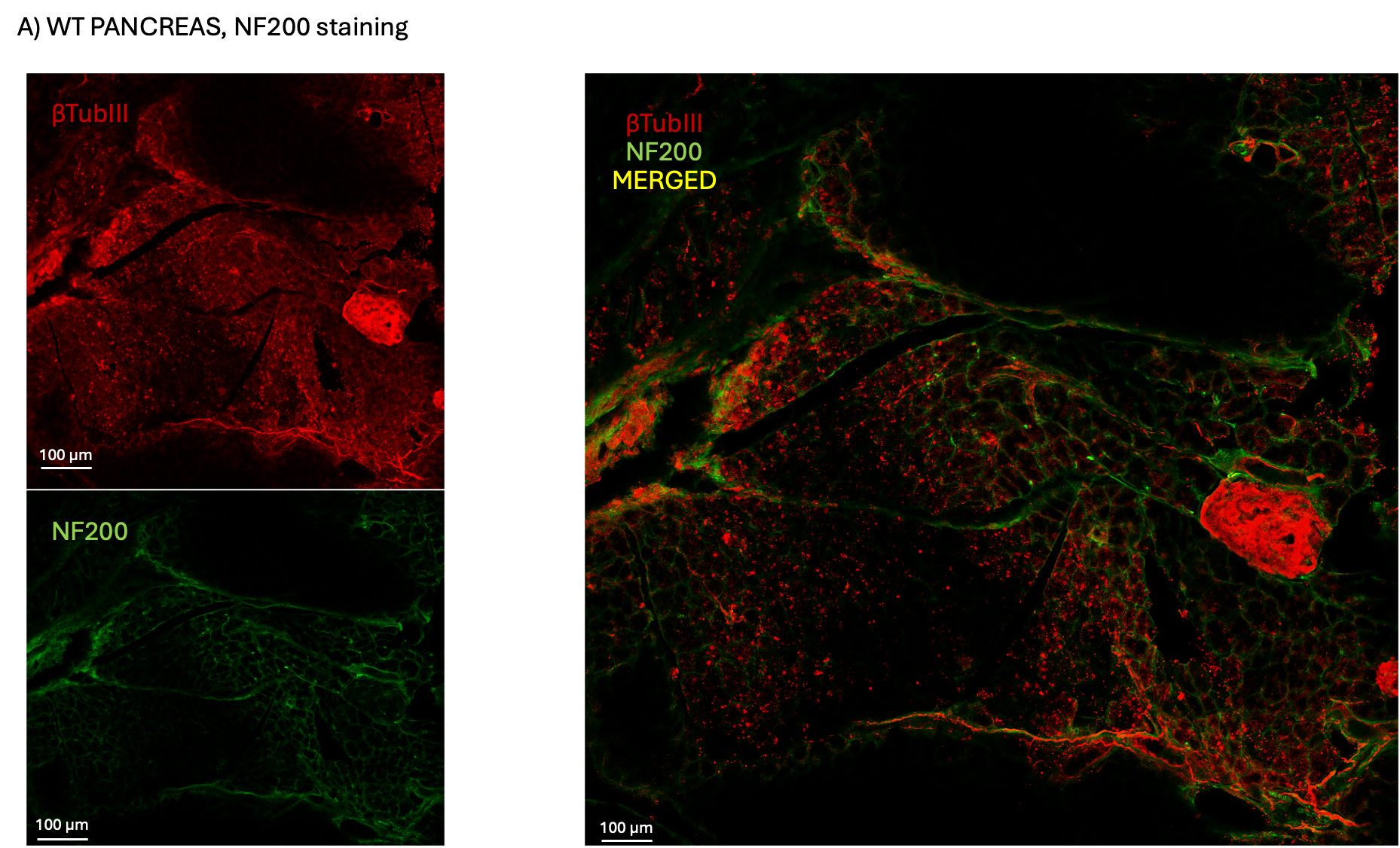


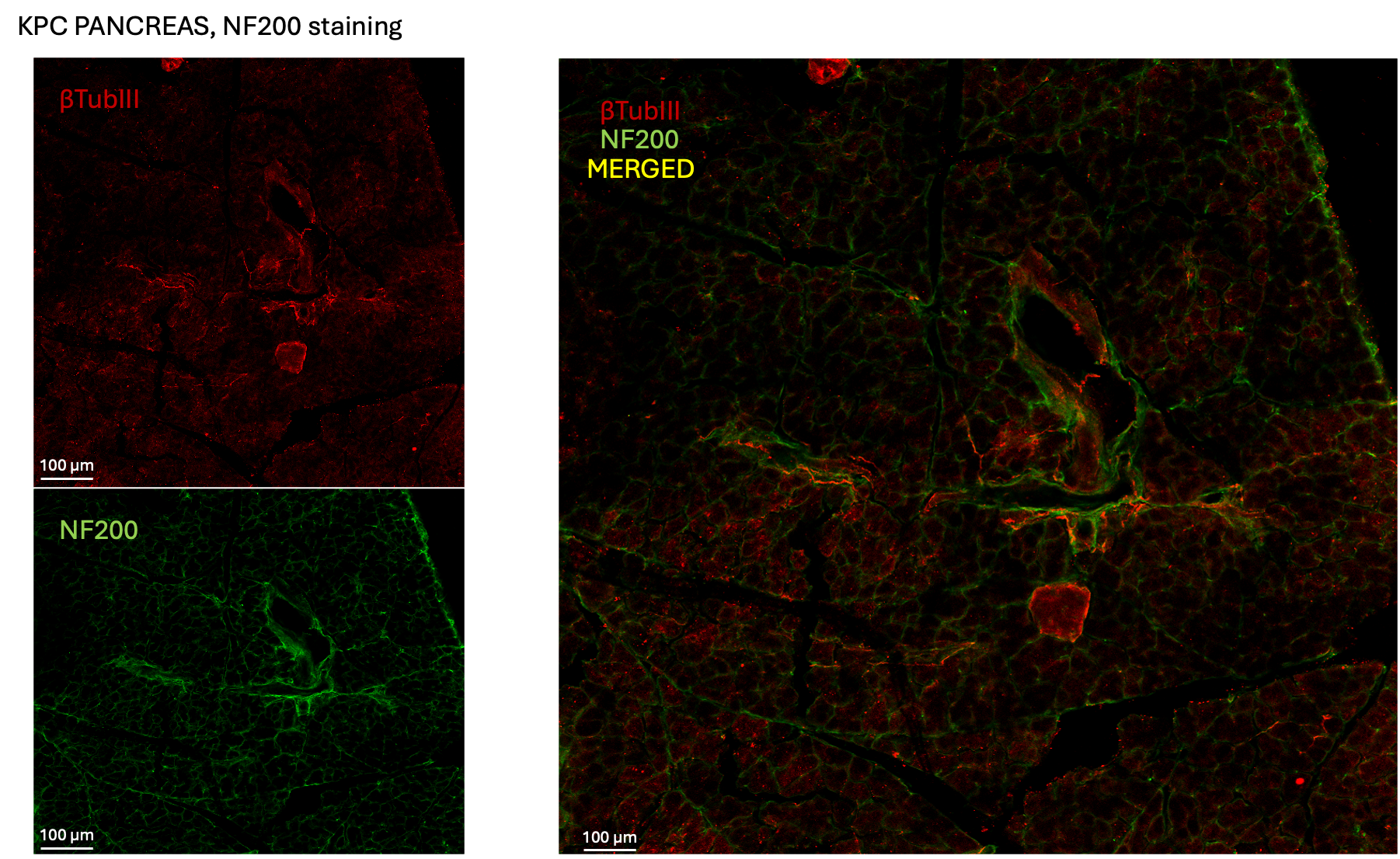


KPC pancreas zoom, NF200 staining


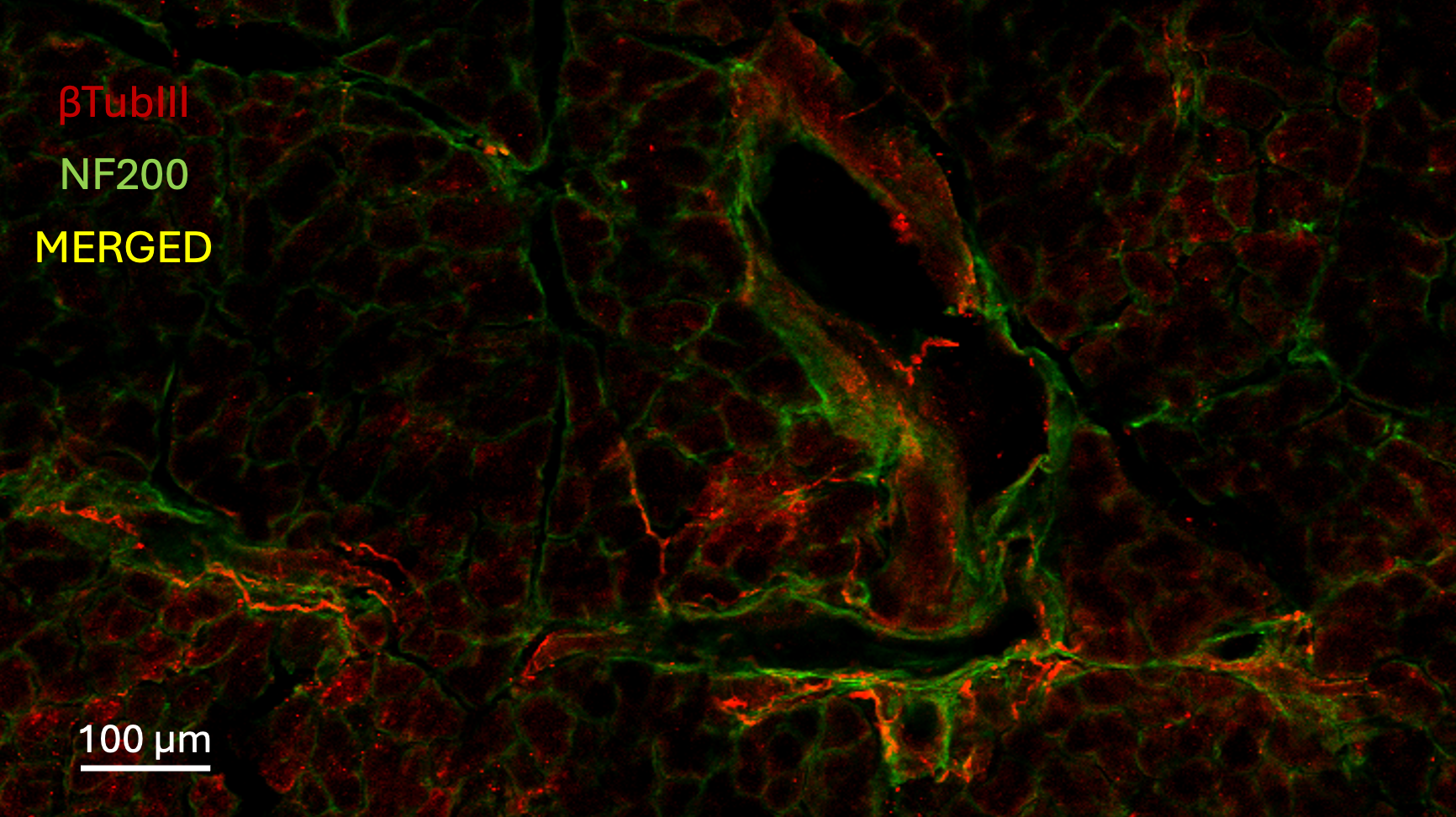


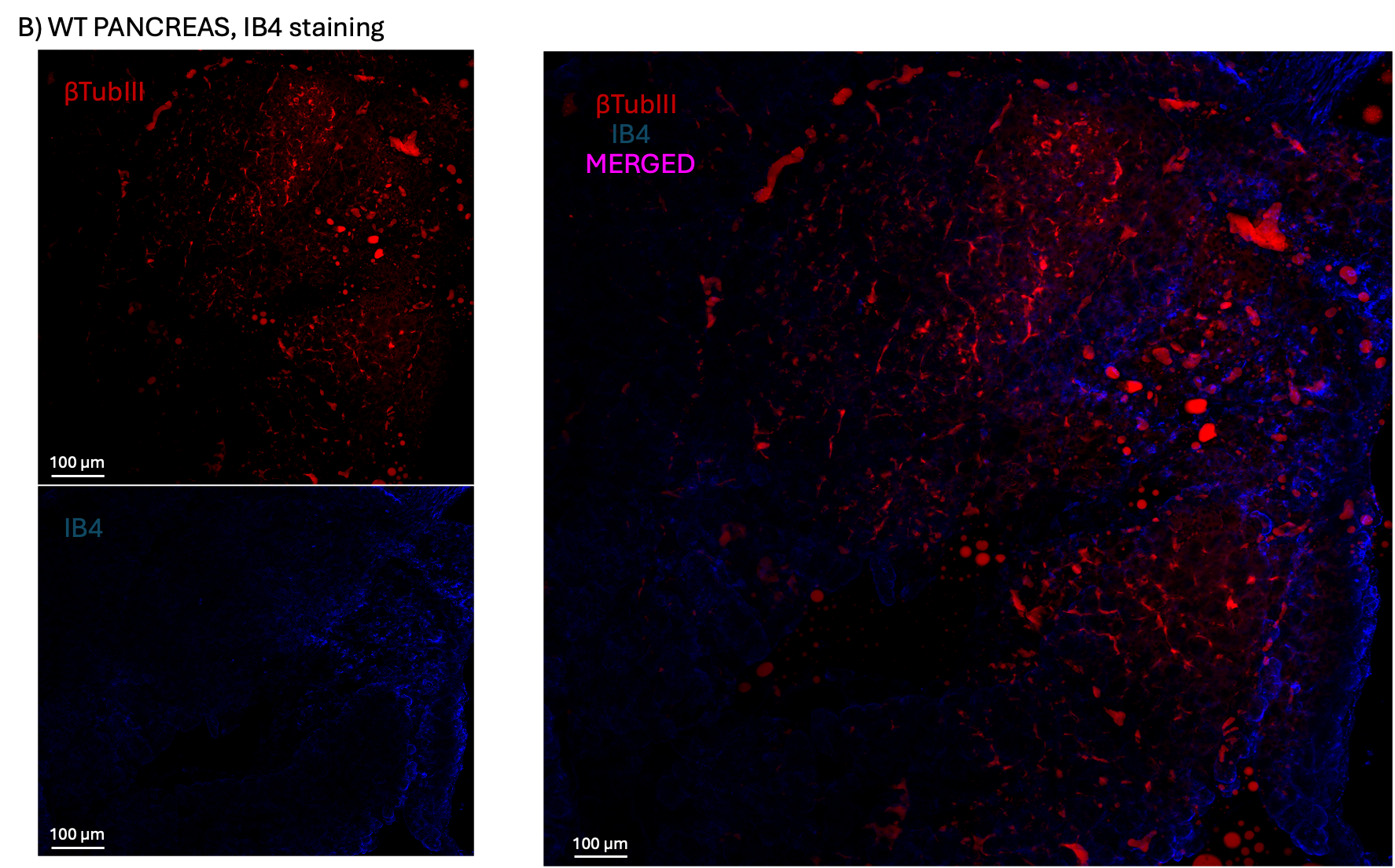


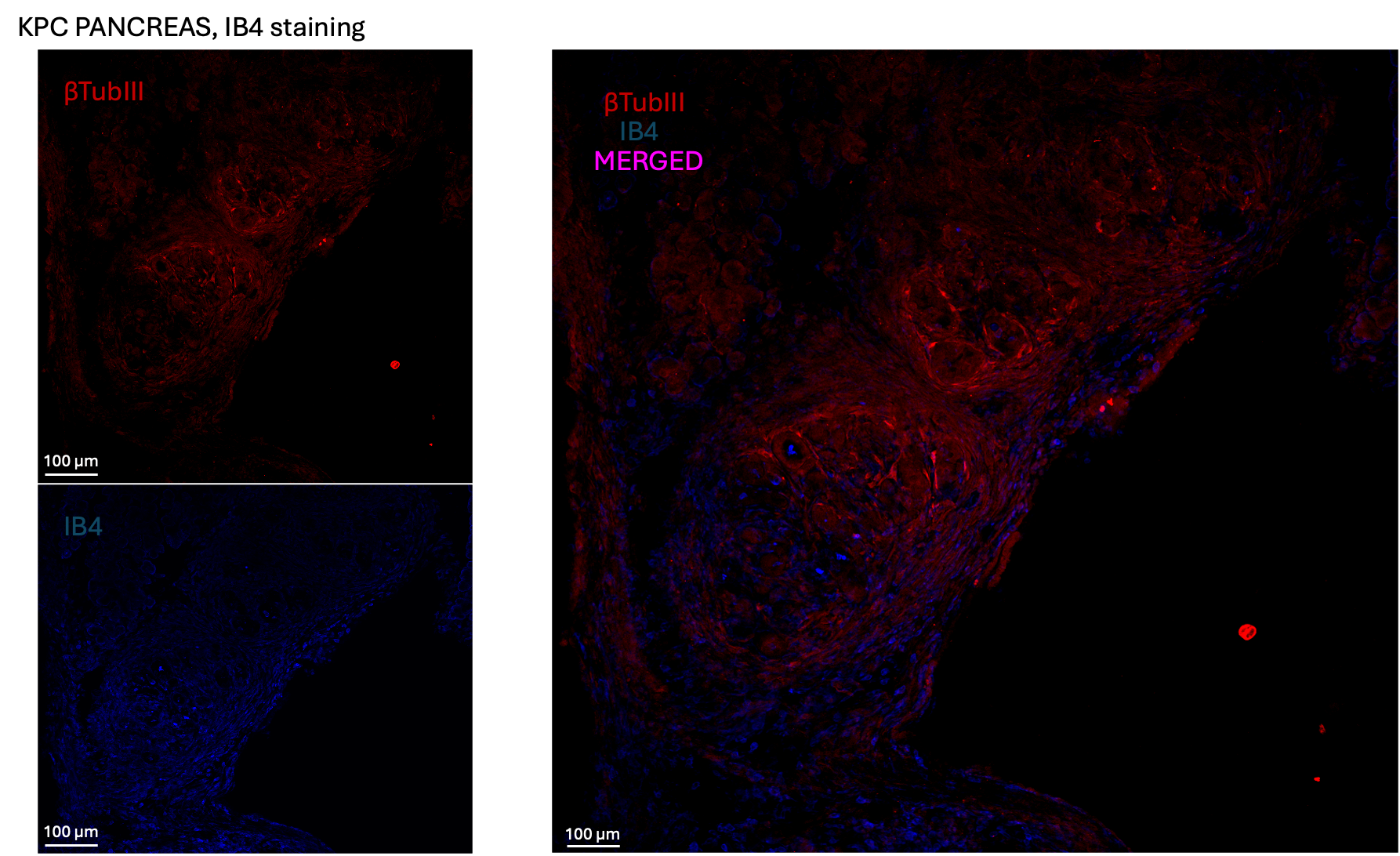


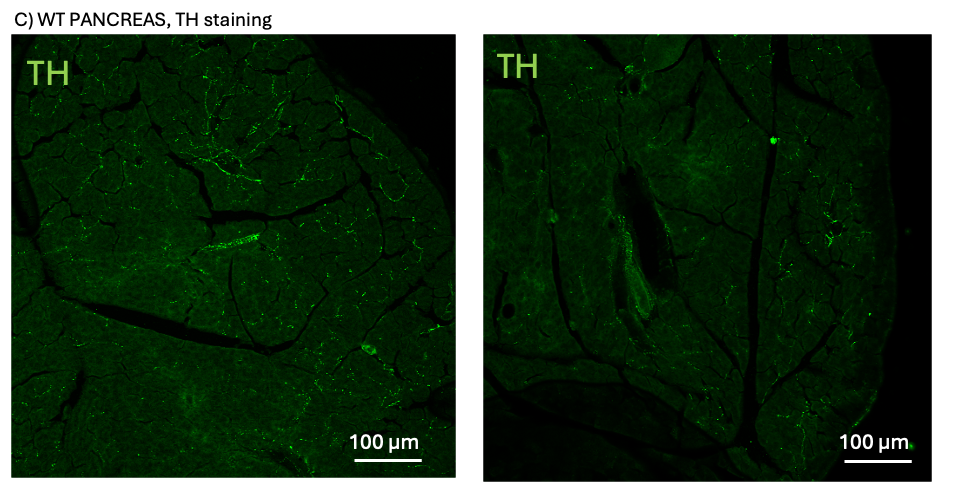


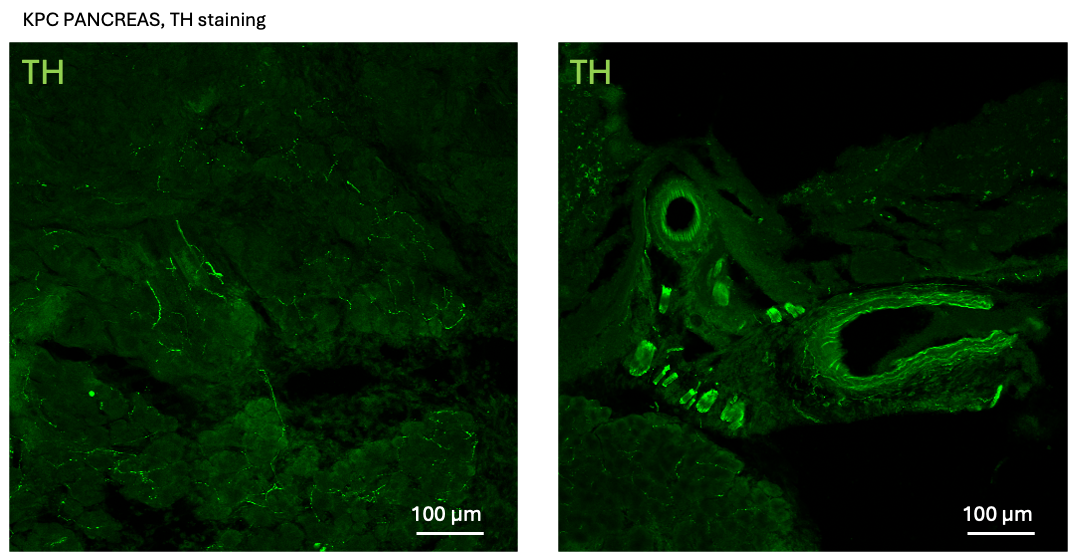


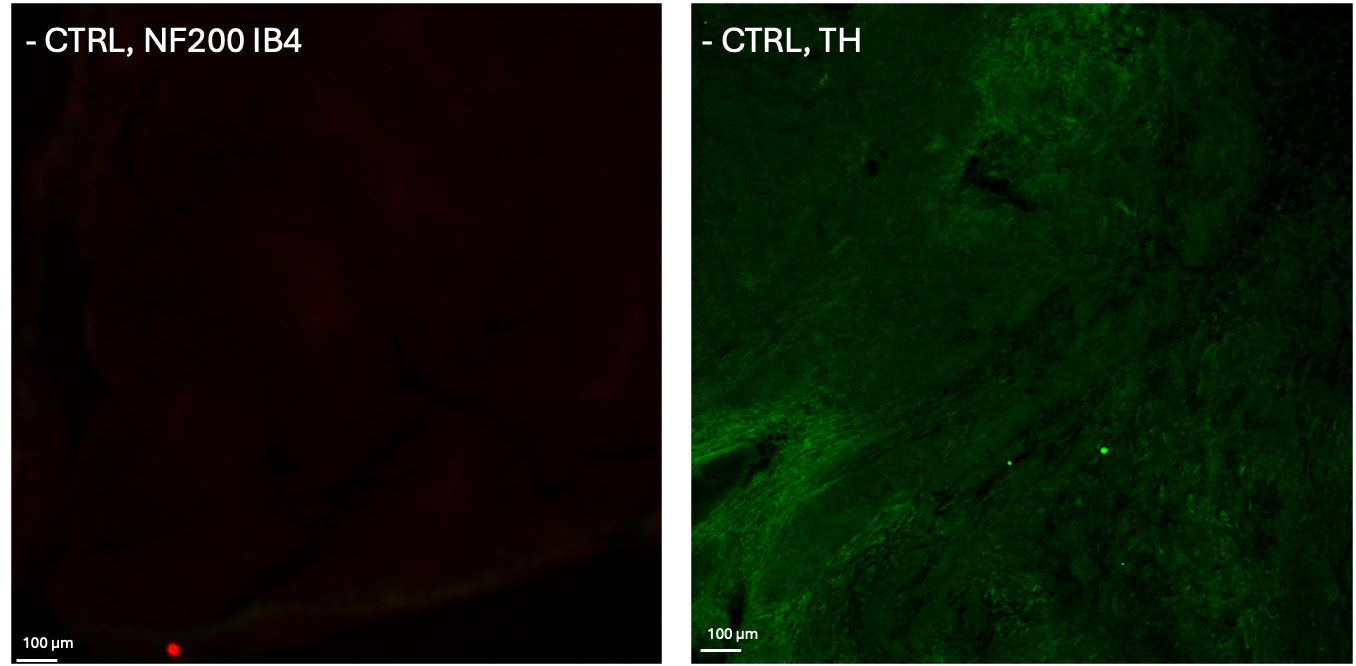


Fig. S4. Representative images of sensory neuron subpopulation marker immunostaining on pancreatic slices. 30µm thick pancreatic tissue from both KPC and WT animals were stained for β-Tub III (for axon localization) and each of the selected DRG sensory neurons subpopulation marker (A: NF200 for “NF”, green; B: IB4 for “NP”, blue; and C: TH for “TH”, green). Axonal localization was found in both tumour and normal pancreas only for NF and TH subtypes. Scalebar = 100 µm.


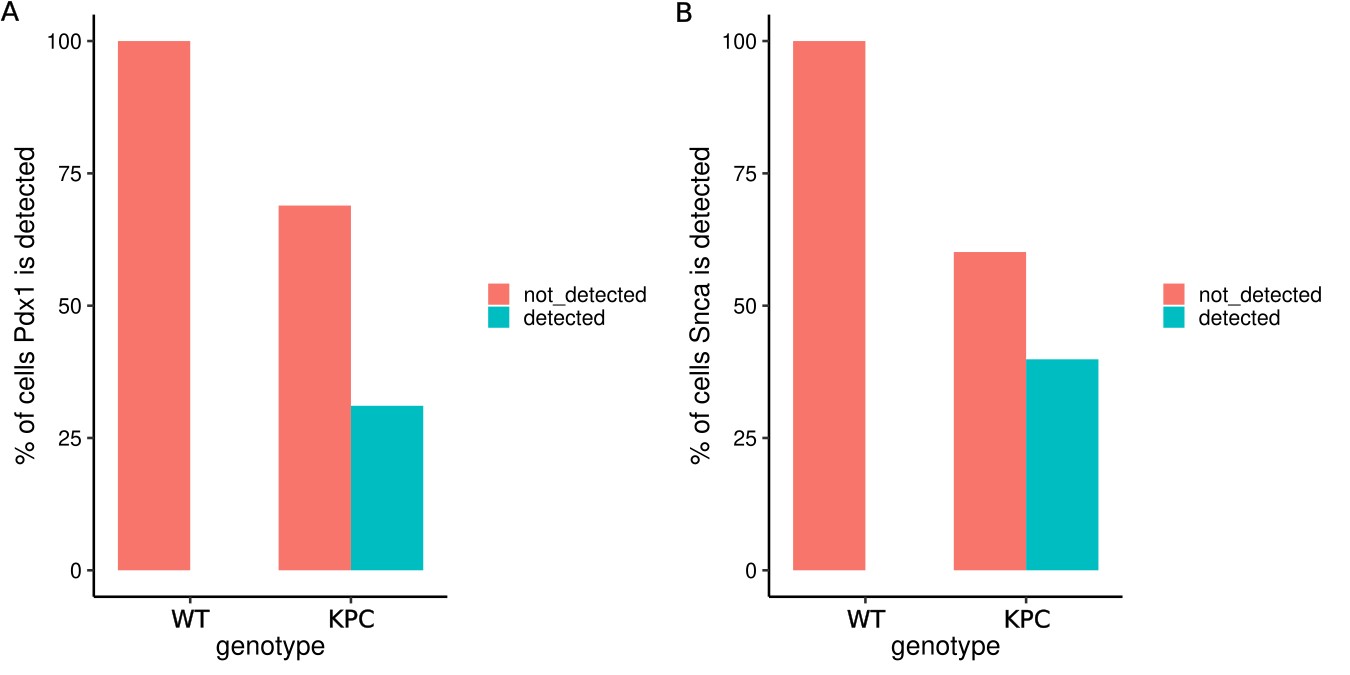


Fig. S5. A: Percentage of WT and KPC DRG cells expressing Pdx1 5’UTR. Pdx1 5’UTR was detected in 31% of KPC cells, predominantly in NF DRGs, while it was not detected in WT DRGs. B: Percentage of WT and KPC DRG cells expressing Snca. Snca was detected in 40% of KPC cells, predominantly in NF DRGs, while it was not detected in WT DRGs


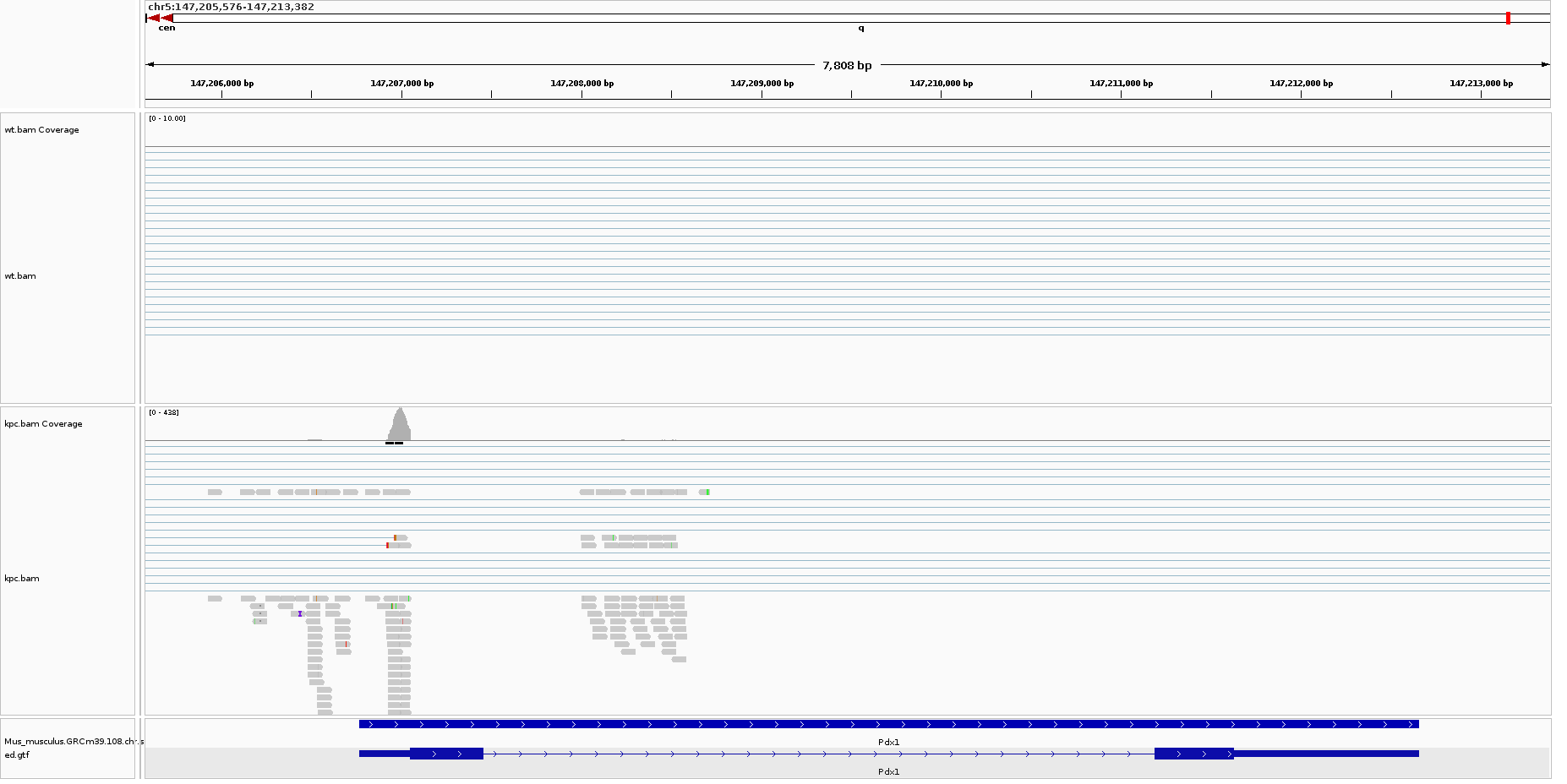


Fig S6. IGV snapshot of neurons (rows) whose Pdx1 5’UTR count > 10. Evidence of reads mapping particularly on the 5’ UTR of Pdx1.


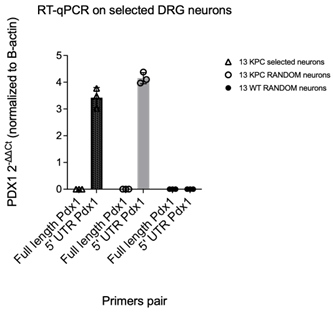


Fig. S7. Barplot of RT-qPCR validation of gene of interest (*Pdx1*) across 3 sample types as a pool of selected DRG neurons from the scRNAseq library (*Pdx1* UMI ≥10; 13 cells from KPC mice 1,2 and 3). Controls were 13 randomly picked DRG neurons from a different animal (mouse KPC4, positive control) and from a WT animal (mouse WT2, negative control). We could not detect the full length *Pdx1* transcript, while we detected the Pdx1 5’UTR fragment in both the pools from KPC animals. Data are reported as the mean of ∆∆Ct in 3 technical replicates, normalized to *β-actin*. The positive control consisted of 10ng cDNA from the pancreas of a WT C57BL/6J mouse. Normalization was performed using the values for *Pdx1 5'UTR* and *β-actin* obtained from this positive control. Error bars represent mean ± SD. No statistical test was applied, as *Pdx1* full length transcripts were not detected in either KPC or WT samples, and *Pdx1 5’UTR* transcripts were detected in KPC samples only; individual points represent technical replicates.


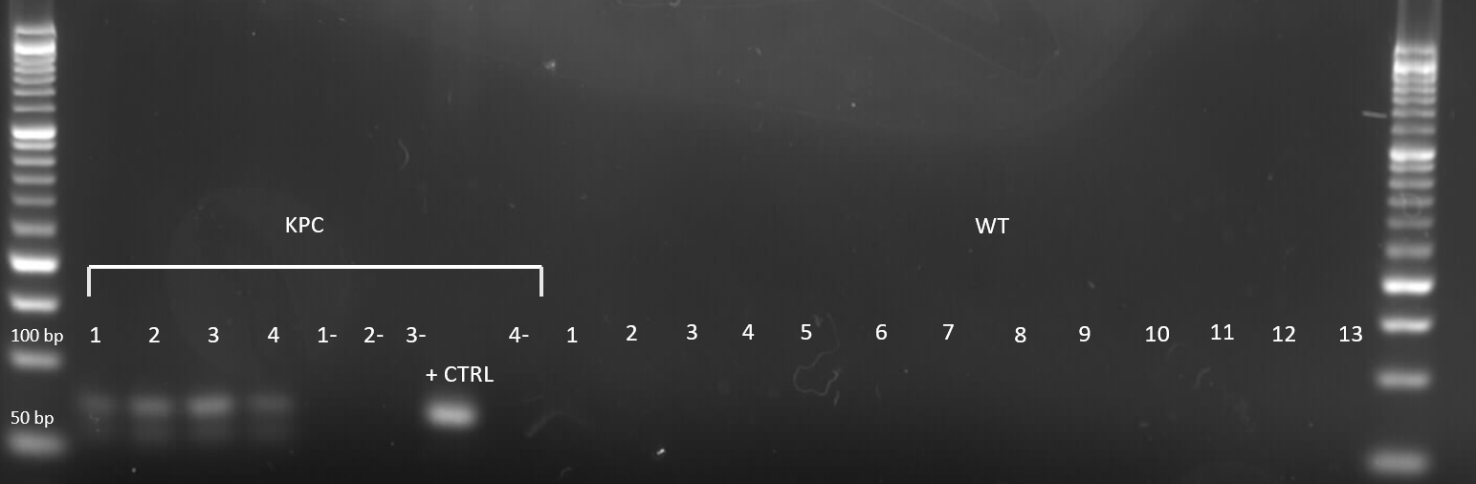


A

Fig. S8. A: 2.5% agarose gel in TBE 0.5x for *Pdx1 5’UTR* transcript from pooled DRG neurons from within scRNAseq library. The *Pdx1 5’UTR* amplicon (71bp) is found in pooled KPC DRG neurons only. cDNA negative controls (1,2,3 and 4-) without RetroTranscriptase enzyme; positive control is cDNA from KPC organoids, synthesised with a mix of oligodT and random hexamer. KPC 1-4= *Kras^LSL-G12D; p53LoxP; Pdx1-CreER^*  PDAC mice included in scRNAseq dataset; WT 1-13= C57BL/6J mice used as normal pancreas condition, included in scRNAseq dataset. Positive control consisted in 10ng of DNA extracted from a pancreas of a KPC mouse treated with tamoxifen. B: left: 2.5% agarose gel in TBE 0.5x for *Pdx1 5’UTR* transcript from DRG neurons from KPC mice not treated with tamoxifen. Pdx1 5’ UTR and Pdx1 5’UTR - Tg Cre signals were absent. Positive control was 10 ng DNA from KPC mouse pancreas treated with tamoxifen. B, right: Pdx1 full length primer pairs 1 and 2 tested in cDNA from Normal Pancreas (NP) organoids indicating that they are indeed functional. Positive control was cDNA from C57BL/6J mouse pancreas.


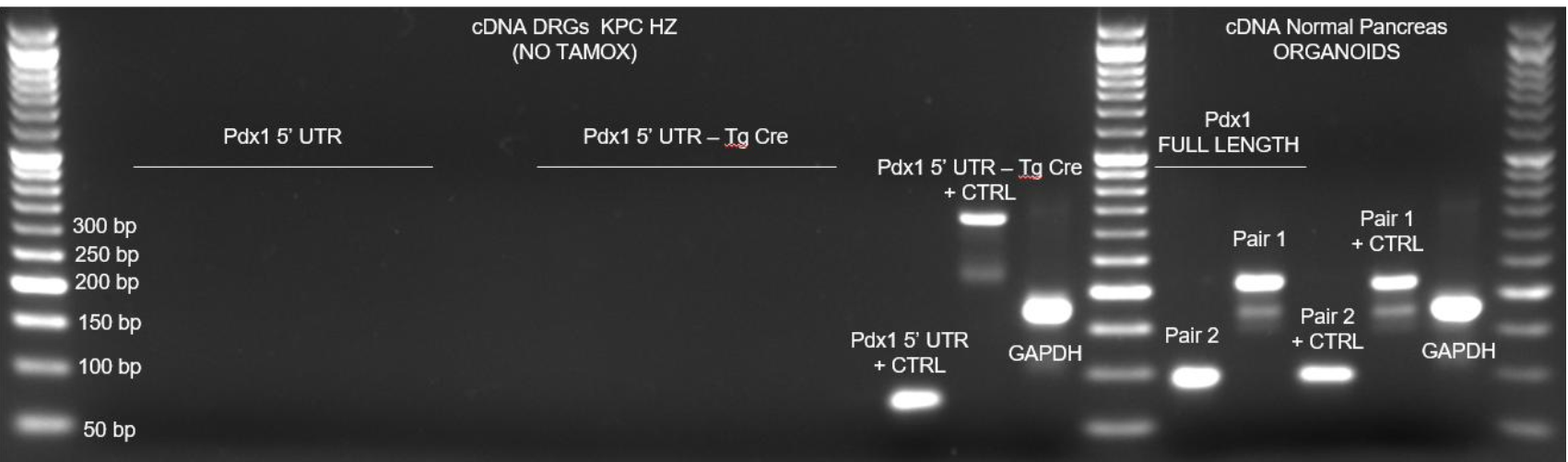


B


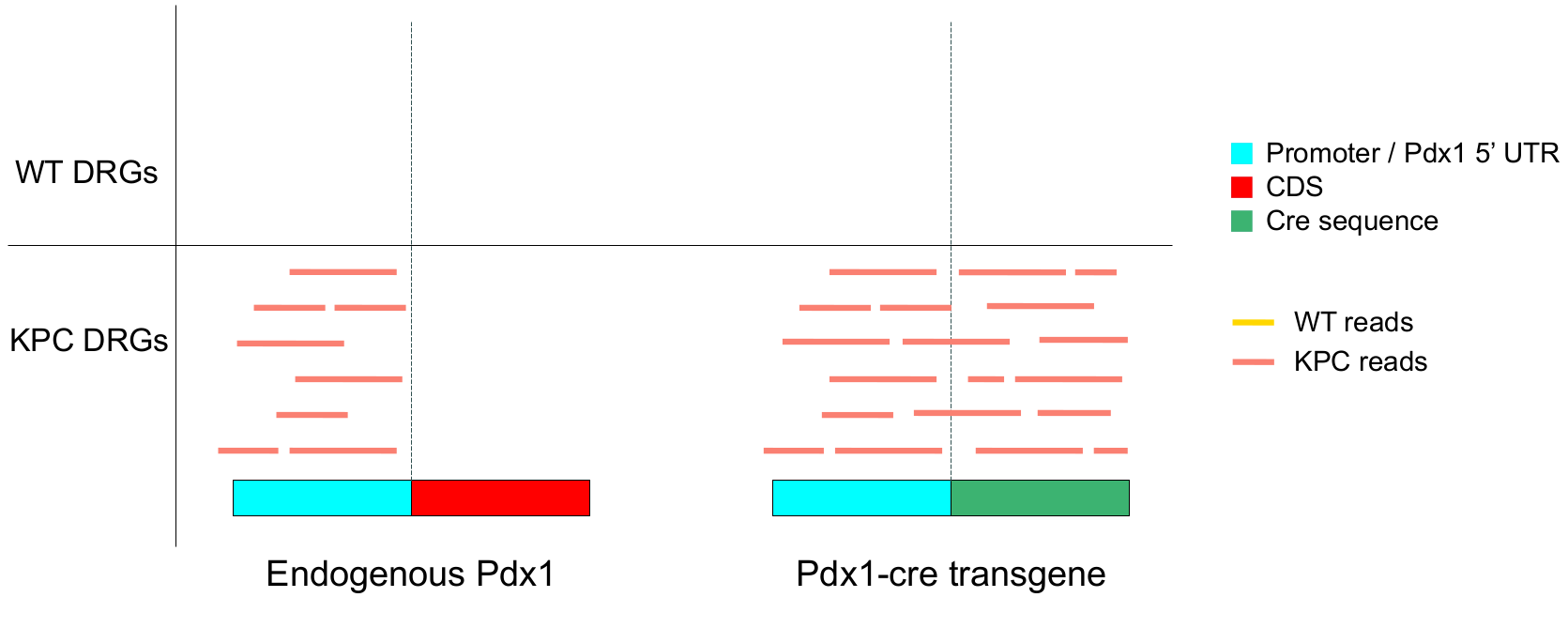


Fig. S9. Schematic cartoon of scRNAseq reads from sequenced KPC and WT DRG neurons innervating the pancreas (related to Fig. S4).


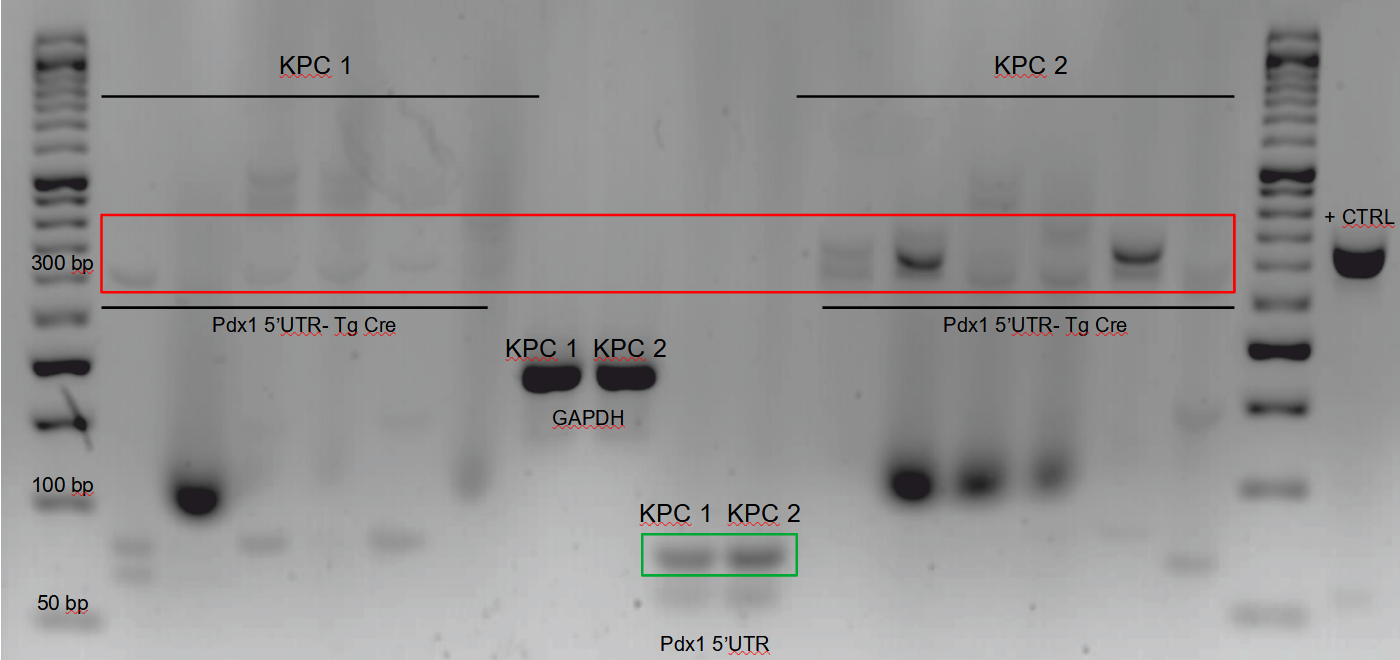


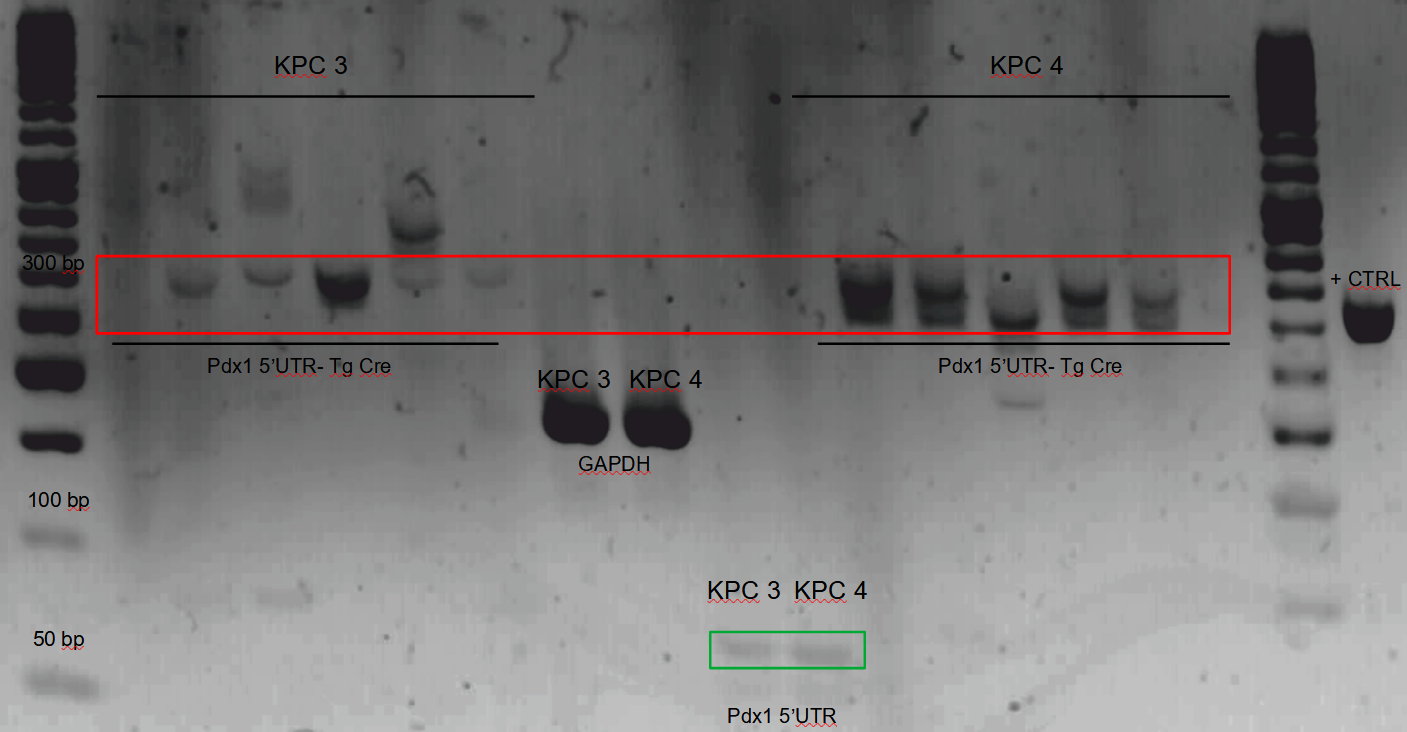


Fig. S10. RT-PCR of PDX1 5’UTR-Tg Cre and Pdx1 5’ UTR sequences in DRG innervating pancreatic tissue from KPC mice. RNA was extracted from 20 DRG neurons randomly picked from each animal within our scRNAseq dataset. RT-PCR products were run on a 2.5% agarose gel in TBE 0.5x. *Pdx1 5’UTR* amplicon (71bp, green box) is found in pooled KPC DRG neurons (as also demonstrated in Figure S8A) alongside the *Pdx1 5’UTR-Tg Cre* transgene amplicon (327 bp, redbox); Positive control is 10 ng DNA extracted from pancreatic tissue of a KPC animal treated with tamoxifen. Gel images are shown in negative to accentuate the weak ethidium bromide staining of the low molecular weight bands in the lower gel.


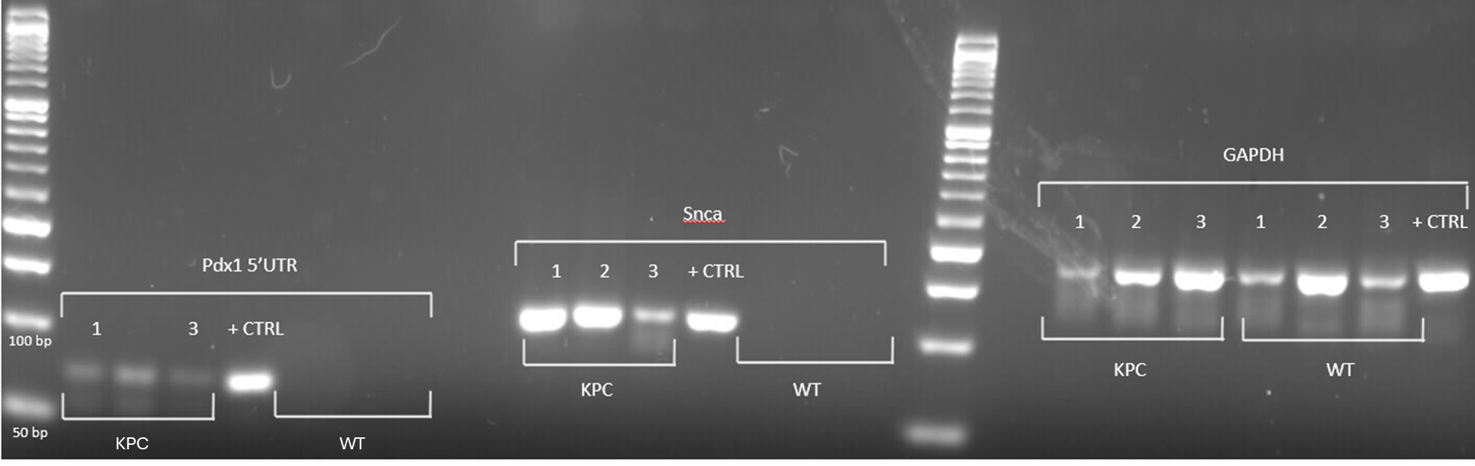


Fig. S11. 2.5% agarose gel in TBE 0,5x for *Pdx1 5’UTR* transcript and *Snca* in RT-qPCR in EVs isolated from blood. *Pdx1 5’UTR* amplicon (71 bp) and *Snca* amplicon (112 bp) in EVs isolated from KPC blood only (N=3). *GAPDH* (173 bp) is reported here as a positive control for successful EVs isolation. KPC= EVs isolated from blood of *Kras^LSL-G12D+/+; p53LoxP; Pdx1-CreER^ ;* WT*=* EVs isolated from blood of *Kras^LSL-G12D-/-; p53LoxP; Pdx1-CreER^* siblings. Both mouse types were treated with tamoxifen. Positive control for Snca consisted of cDNA from 10^6^ cortical neurons from a C57BL/6J WT mouse, while positive control for Pdx1 5’UTR consisted of 10 ng of total DNA extracted from the pancreas of a KPC mouse treated with tamoxifen.


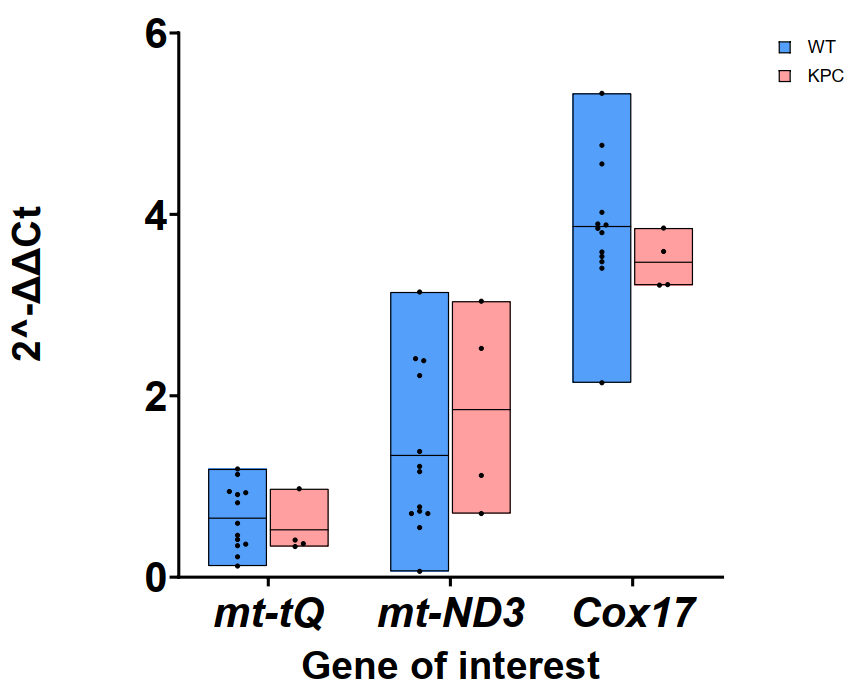


Normalized on MCN

Normalized on genomic DNA

Fig. S12. Boxplot of RT-qPCR validation of genes of interest (*mttQ, Cox17* and *mt-ND3*) showing differential distribution across all animals tested (N= 4 KPC, N= 13 WT). Samples were treated as technical replicates, as 20 cells were pooled for each animal starting from lysates sent to scRNA seq analysis. *mttQ* and *mt-ND3*: normalized to total mitochondrial DNA copy number (MCN) using *β2-microglobulin* (genomic DNA) and *mt-RNR2* (mitochondrial DNA) as reference genes to calculate absolute mitochondrial copy number within each sample. For *Cox17*, positive control consisted of cDNA from 13 DRG neurons (1 neuron per animal) randomly selected from each WT animal included in the scRNAseq dataset, normalized using *β2-microglobulin* (genomic DNA) only. MCN = Mitochondrial Copy Number. KPC = *Kras^LSL-G12D; p53LoxP; Pdx1-CreER^*  KPC mice 1-4 included in scRNAseq dataset; WT = C57BL/6J mice 1-13 used as normal pancreas condition, included in scRNAseq dataset (t-test, p = ns).


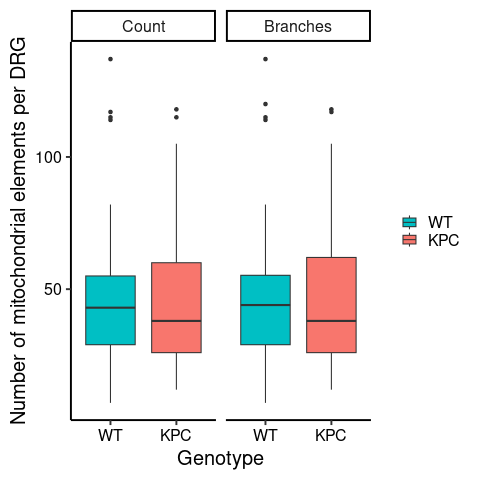


Fig S13. Mitochondrial count and number of branches comparing FB+ WT and KRAS DRGs at the overall population level. No significant differences were identified (t-test, p = ns). Boxplots show the median (center line), interquartile range (box), and whiskers extending to 1.5×IQR; individual points represent technical replicates or DRG neurons.

Table S1. Primers used in this study

| **Target** | **FW (5'->3')** | | **RV (5'->3')** |
| --- | --- | --- | --- |
| Snca | GGCATTTCATAAGCCTCACTGC | | CCACTGGCTTTGTCAAGAAGG |
| mt-Tq | CTAGGACAATAGGAATTGAACC | | TAGGATAAGGTGTTTAGGTAGCA |
| mt-ND3 | TCGACCCTACAAGCTCTGC | | CATGGTAGTGGAAGTAGAAGAGC |
| Cox17 | ACTGAAGCCCTGCTGTGC | | CCCTCATGCACTCCTTGTGG |
| Pdx1 full length  (PAIR 1) | CTCCCTTTCCCGTGGATGAA | | GGTCAAGTTCAACATCACTGCC |
| Pdx1 full length  (PAIR 2) | ATGAAATCCACCAAAGCTCACG | | GGCAGTACGGGTCCTCTTG |
| Pdx1 5' UTR | AACTGTCAAAGCGATCTGGG | | CTACAAGCCAGGCCTTAGG |
| Pdx1 5' UTR–Tg Cre | AACTGTCAAAGCGATCTGGG | | CGAACATCTTCAGGTTCTGC |
| GAPDH | ATCTTCTTGTGCAGTGCCAGCCTCGTC | | GAACATGTAGACCATGTAGTTGAGGTCAATGAAGG |
| B actin | CACTGTCGAGTCGCGTCC | | TCATCCATGGCGAACTGGTG |
| β2-microglobulin | ATGGGAAGCCGAACATACTG | | CAGTCTCAGTGGGGGTGAAT |
| mt-RNR2 | CTAGAAACCCCGAAACCAAA | | CCAGCTATCACCAAGCTCGT |
|  | |  |  |

Table S2. See attached Table S2 (7430 rows x 12 column)
